# Impact of Cardiac Arrest Centers on the Survival of Patients With Nontraumatic Out‐of‐Hospital Cardiac Arrest: A Systematic Review and Meta‐Analysis

**DOI:** 10.1161/JAHA.121.023806

**Published:** 2021-12-20

**Authors:** Jun Wei Yeo, Zi Hui Celeste Ng, Amelia Xin Chun Goh, Jocelyn Fangjiao Gao, Nan Liu, Shao Wei Sean Lam, Yew Woon Chia, Gavin D. Perkins, Marcus Eng Hock Ong, Andrew Fu Wah Ho, the National Targeted Temperature Management Workgroup, Shiang‐Hu Ang, Ruth Weixian Chen, Enoch Hin Kei Chan, Ee Ling Goh, Vui Kian Ho, Hong Khai Lau, Eng Kiang Lee, Benjamin Sieu‐Hon Leong, Jia Hao Lim, Shir Lynn Lim, Julian Kenrick Xingyuan Loh, Jimmy Heng Ann Ong, Kah Hua Peck, Daniel Yong Jing Quek, Christopher Ying Hao Seet, Shobbit Swarup, Thon Hon Yong

**Affiliations:** ^1^ Yong Loo Lin School of Medicine National University of Singapore Singapore; ^2^ Centre for Quantitative Medicine Duke‐NUS Medical School National University of Singapore Singapore; ^3^ Health Services Research Centre SingHealth Duke‐NUS Academic Medical Centre Singapore; ^4^ Department of Cardiology Tan Tock Seng Hospital Singapore; ^5^ Warwick Medical School University of Warwick Coventry United Kingdom; ^6^ Department of Emergency Medicine Singapore General Hospital Singapore; ^7^ Health Services & Systems Research Duke‐NUS Medical School Singapore; ^8^ Pre‐Hospital and Emergency Research Centre Health Services and Systems Research Duke‐NUS Medical School Singapore

**Keywords:** cardiac arrest, cardiac arrest center, heart arrest, resuscitation, Cardiopulmonary Arrest, Cardiopulmonary Resuscitation and Emergency Cardiac Care

## Abstract

**Background:**

The role of cardiac arrest centers (CACs) in out‐of‐hospital cardiac arrest care systems is continuously evolving. Interpretation of existing literature is limited by heterogeneity in CAC characteristics and types of patients transported to CACs. This study assesses the impact of CACs on survival in out‐of‐hospital cardiac arrest according to varying definitions of CAC and prespecified subgroups.

**Methods and Results:**

Electronic databases were searched from inception to March 9, 2021 for relevant studies. Centers were considered CACs if self‐declared by study authors and capable of relevant interventions. Main outcomes were survival and neurologically favorable survival at hospital discharge or 30 days. Meta‐analyses were performed for adjusted odds ratio (aOR) and crude odds ratios. Thirty‐six studies were analyzed. Survival with favorable neurological outcome significantly improved with treatment at CACs (aOR, 1.85 [95% CI, 1.52–2.26]), even when including high‐volume centers (aOR, 1.50 [95% CI, 1.18–1.91]) or including improved‐care centers (aOR, 2.13 [95% CI, 1.75–2.59]) as CACs. Survival significantly increased with treatment at CACs (aOR, 1.92 [95% CI, 1.59–2.32]), even when including high‐volume centers (aOR, 1.74 [95% CI, 1.38–2.18]) or when including improved‐care centers (aOR, 1.97 [95% CI, 1.71–2.26]) as CACs. The treatment effect was more pronounced among patients with shockable rhythm (*P*=0.006) and without prehospital return of spontaneous circulation (*P*=0.005). Conclusions were robust to sensitivity analyses, with no publication bias detected.

**Conclusions:**

Care at CACs was associated with improved survival and neurological outcomes for patients with nontraumatic out‐of‐hospital cardiac arrest regardless of varying CAC definitions. Patients with shockable rhythms and those without prehospital return of spontaneous circulation benefited more from CACs. Evidence for bypassing hospitals or interhospital transfer remains inconclusive.

**Nonstandard Abbreviations and Acronyms**
CAC: cardiac arrest centers
OHCA: out‐of‐hospital cardiac arrest
ROSC: return of spontaneous circulation
TTM: targeted temperature management

**Clinical Perspective**

**What Is New?**

- There is uncertainty over the role of cardiac arrest centers (CACs) in the care of out‐of‐hospital cardiac arrest (OHCA), and the 2020 International Liaison Committee on Resuscitation guidelines previously recommended with low certainty that patients with OHCA should be transported to a CAC, partly based on a systematic review on the topic.
- Treatment of nontraumatic patients with OHCA at CACs was associated with significantly improved survival and neurological outcomes, and these findings persisted even when using varying definitions of CAC (eg, high‐volume centers).
- The treatment effect was more pronounced among patients with OHCA with shockable rhythm and those without prehospital return of spontaneous circulation.

**What Are the Clinical Implications?**

- The current updated systematic review and meta‐analysis provided an upgraded level of evidence (Grading of Recommendations, Assessment, Development, and Evaluation level of evidence: moderate) in support of transport of nontraumatic patients with OHCA to CACs, and patients who would likely benefit most are those with shockable rhythms and those without prehospital return of spontaneous circulation.
- Regionalized care for patients with OHCA has the potential to improve outcomes, but transport policies that involve bypassing the nearest hospital for CACs or for interhospital transfer from non‐CACs to CACs need further studies.

Out‐of‐hospital cardiac arrest (OHCA) is the most time‐critical medical emergency^1^, ^2^, ^3^ and exerts a tremendous disease burden.^4^ The post–cardiac arrest syndrome, a consequence of whole‐body ischemia‐reperfusion injury with devastating multiorgan involvement, is a significant contributor to poor outcomes among OHCA survivors, for which complex multidisciplinary care is required.^5^, ^6^, ^7^ Postresuscitation care has been suggested to be the fifth link in the chain of survival concepts, and a component of an integrated emergency care network comprising community first responders, emergency medical services (EMS), and hospitals aiming to provide quality care to patients with OHCA. Despite advances in therapeutics such as targeted temperature management (TTM), mechanical circulatory support, and neuroprognostication, urgent questions remain pertaining to how best to organize hospitals and emergency care systems to improve access to quality care and clinical outcomes.^8^

The recent 2020 guidelines from the International Liaison Committee on Resuscitation recommended with low certainty^9^, ^10^, ^11^ that patients with OHCA should be transported to cardiac arrest centers (CACs). CACs are specialized tertiary institutions, conceptually similar to level 1 trauma centers, and are often high‐volume or regionalized centers treating patients with OHCA with the capability to organize postresuscitation care, including 24/7 access to a cardiac catheterization laboratory for coronary angiography and percutaneous coronary intervention (PCI), TTM, extracorporeal membrane oxygenation, and neuroprognostication among other interventions.^12^, ^13^, ^14^ However, although there has been evidence for the effectiveness of each individual intervention in variable settings,^15^, ^16^, ^17^, ^18^, ^19^ evidence for the benefit of CACs in treating patients with OHCA remain inconclusive. This is in part because CACs, which provide a complex bundle of interventions, have been poorly defined,^10^, ^20^ and similar institutions described in published literature may range from exhibiting only a few to many of the defining traits of a CAC. This brings about difficulties in statistical analysis and interpretation.

Furthermore, it is unknown which subpopulations benefit more from CACs, defined according to prehospital Utstein variables^21^, ^22^ such as the receipt of bystander cardiopulmonary resuscitation, initial shockable rhythm, or prehospital return of spontaneous circulation (ROSC). A recent cohort study by Chien et al^23^ suggested that the presence of a shockable rhythm modified the benefits of CAC. Kajino et al^24^ also showed significant benefit in patients without, but not for patients with, prehospital ROSC. This knowledge gap is especially pertinent because knowing which patients are likely to benefit from an expensive intervention can guide prioritization of scarce health care resources. This understanding also aids the rational ambulance diversion strategy to bring the right patients to the right destinations.^8^, ^25^

Consequently, this systematic review aimed not only to provide urgently needed evidence for or against treating patients with OHCA at CACs, but also to analyze the impact within predefined subgroups.

## Methods

This systematic review and meta‐analysis adhered to the *Preferred Reporting Items for Systematic Reviews and Meta‐Analyses* guidelines (Table S3).^26^, ^27^ The study protocol had been published in the PROSPERO (International Prospective Register of Systematic Reviews; CRD42021260468). The data that support the findings of this study are available from the corresponding author upon reasonable request.

### Search Strategy

A systematic literature search was performed in MEDLINE, Embase, and Cochrane Central Register of Controlled Trials databases from inception up to March 9, 2021. The search strategy was developed in consultation with a medical information specialist. Keywords and Medical Subject Headings terms such as “cardiac arrest center,” “hospital volume,” “postresuscitation care,” “fifth link,” “out‐of‐hospital cardiac arrest,” and other synonyms were applied in the search strategy to identify relevant articles. Seventy‐one references from the latest systematic review and meta‐analysis on this topic,^10^ including the review itself, were hand searched to identify additional relevant studies. The investigative team, which included several resuscitation scientists, were asked to ascertain if they were aware of additional relevant studies. This process did not surface any study that was not already captured in the search strategy. Articles were viewed through Endnote X9^28^ (Clarivate, Philadelphia, PA) for an article sieve. The search was repeated on June 7, 2021 yielding no additional eligible articles. The detailed search strategy is available in Data S1.

### Inclusion and Exclusion Criteria

An article sieve was conducted by 3 authors (J.W.Y., Z.H.C.N., A.X.C.G.) according to predefined criteria. Each article was reviewed by at least 2 authors blinded to each other’s decision. Disputes were resolved through consensus from the senior author (A.F.W.H.). All studies were filtered through the following inclusion criteria: (1) studies with adult patients with OHCA of nontraumatic cause, (2) studies comparing CAC versus non‐CAC, (3) studies comparing direct transport to CAC versus transfer to CAC, and (4) studies reporting outcomes of interest such as survival to 30 days or hospital discharge and survival to 30 days or hospital discharge with favorable neurological outcome. Good neurological outcome was defined as Cerebral Performance Category 1 or 2, or modified Rankin scale 0, 1, or 2. Both interventional studies, such as randomized clinical trials, and observational studies, such as retrospective or prospective cohorts, were included. Studies with only pediatric patients or with no control group were excluded. Review articles, meta‐analyses, protocols, conference abstracts, letters, commentaries, and editorials were excluded from this review. We excluded studies that were not in the English language and were not accompanied by an English translation.

### Definition of Cardiac Arrest Centers

There was a lack of consensus over the definition of a CAC in the literature.^7^, ^10^, ^12^, ^20^ For example, the Association for Acute CardioVascular Care of the European Society of Cardiology described cardiac arrest centers^12^ as specialized institutions offering all recommended treatment options for patients with OHCA, including access to a coronary angiography laboratory with 24/7 PCI capability, TTM, extracorporeal membrane oxygenation, mechanical ventilation, and neurological prognostication. On the other hand, the German Resuscitation Council accreditation process required CACs to have standard operating procedures for communication with EMS and quality of care assessments in addition to 24/7 PCI, TTM, and intensive care capabilities.^29^ Using a strict definition of CAC, only institutions with the capability for 2 or more of the above interventions and explicitly referred to by study authors as CACs or synonymous terms, such as critical care medical center, tertiary heart center, cardiac receiving center, and regional center, were accepted. PCI‐capable hospital alone was not accepted as a term synonymous with CAC. Having the capability for only 1 of the above interventions was also considered insufficient, because a single intervention cannot constitute an intervention bundle. To account for differences in defining CACs, sensitivity analyses were conducted using less strict definitions, accepting terms like high‐volume centers and centers with improved postresuscitation care including before‐and‐after study designs.

### Statistical Analysis

Data on general article information (author, year, country), baseline demographics (age, sex, witnessed arrest, initial shockable rhythm, prehospital ROSC), and outcomes of interest (survival to 30 days or hospital discharge with good neurological outcome, survival to 30 days or hospital discharge) were abstracted by 3 authors (J.W.Y., Z.H.C.N., A.X.C.G.). The data abstraction process was blinded among the authors, who used a predetermined data collection form. Disputes were resolved through consensus from the senior author (A.F.W.H.). Adjusted odds ratio (aOR) and crude odds ratio (OR) for binary outcomes were abstracted from each article. Where incremental or hierarchical statistical models were presented, the OR adjusted for the maximum number of covariates was extracted. Where multiple statistical approaches were presented (eg, multivariable modeling and propensity‐score matching) in the same study, we considered the approach used in the primary analysis. When unavailable, OR and 95% CI were calculated for articles reporting summary data using 2×2 contingency tables.

Conventional pairwise meta‐analyses were performed. Given the high known concordance^30^ between the outcomes of survival to 30 days and survival to hospital discharge, the decision was made in consensus with all study authors to pool both outcomes, which was deemed sufficient to demonstrate improvement in short‐term OHCA outcomes, if any, consistent with the Core Outcome Set for Cardiac Arrest.^31^ The aORs were preferentially analyzed over ORs, because the estimates represent less bias caused by confounding. A DerSimonian‐Laird random‐effects model with inverse variance weights was applied regardless of heterogeneity because of expected between‐study variations in population and interventions. Sensitivity analyses were performed for wider definitions of a CAC, including CACs defined as strictly explicit CACs and high‐volume centers, or CACs and centers with improved postresuscitation care. Further sensitivity analyses applying fixed‐effects models to the above were also performed. Heterogeneity was assessed using the *I* ^2^ statistic with 25%, 50%, and 75% thresholds for low, moderate, and high levels of heterogeneity, respectively. To account for heterogeneity, subgroup analyses were performed to compare studies measuring outcomes by hospital discharge versus 30 days, and also among all included studies for predefined, clinically important Utstein variables: initial shockable rhythm and presence of prehospital ROSC whenever possible. All analyses were performed using Review Manager (RevMan 5.4) software package^32^ by the Cochrane Collaboration. Two‐tailed statistical significance was set at *P*<0.05. Publication bias was assessed through visually inspecting funnel plots when 10 or more studies reported an outcome. The quality of observational studies was evaluated on the Newcastle‐Ottawa scale,^33^ and randomized clinical trial risk of bias was evaluated using the Cochrane Risk of Bias 2^34^ tool. Two authors (J.W.Y., Z.H.C.N.) evaluated each article using the Newcastle‐Ottawa scale or Cochrane Risk of Bias 2 tool, and disputes were resolved through consensus from the senior author (A.F.W.H.). The certainty of evidence was assessed using the Grading of Recommendations Assessment, Development, and Evaluation approach (Table S4) and the GRADEpro Guideline Development Tool (Evidence Prime, McMaster University).^35^, ^36^

## Results

### Literature Retrieval and Summary of Included Articles

**Figure 1:**
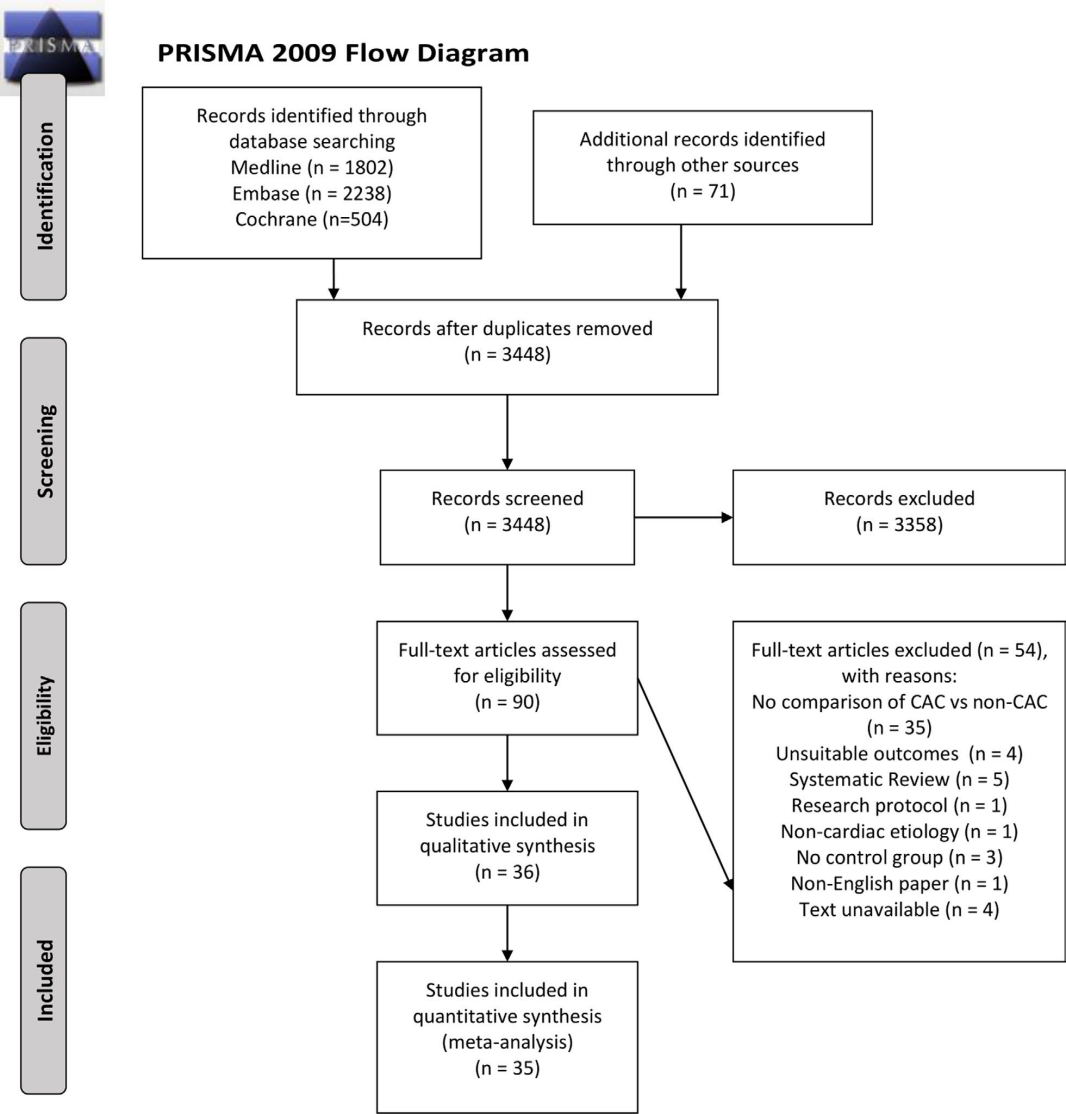
Preferred Reporting Items for Systematic Reviews and Meta‐Analyses (PRISMA) flowchart. CAC indicates cardiac arrest center.

The database search yielded 4544 articles. There were 1093 duplicate articles removed, and 3358 articles were excluded on the basis of their titles and abstracts. A further 54 articles were excluded upon full‐text review. The κ value measuring interrater reliability was 0.75 when reviewing the title and abstracts and 0.9 for full‐text review. Finally, 36 studies^20^, ^23^, ^24^, ^25^, ^37^, ^38^, ^39^, ^40^, ^41^, ^42^, ^43^, ^44^, ^45^, ^46^, ^47^, ^48^, ^49^, ^50^, ^51^, ^52^, ^53^, ^54^, ^55^, ^56^, ^57^, ^58^, ^59^, ^60^, ^61^, ^62^, ^63^, ^64^, ^65^, ^66^, ^67^, ^68^ qualified for analysis. The study selection process and reasons for excluding the 54 studies are illustrated in the Preferred Reporting Items for Systematic Reviews and Meta‐Analyses‐P 2020 flow diagram (Figure 1).

A total of 147 943 patients were included in the 36 studies. Two studies^55^, ^67^ were conducted in Australia, 1 in Canada^66^, 1 in the Czech Republic,^60^ 2 in Denmark,^47^, ^53^ 1 in France,^56^ 5 in Japan,^24^, ^44^, ^50^, ^58^, ^64^ 8 in South Korea,^40^, ^42^, ^46^, ^48^, ^49^, ^51^, ^62^, ^63^ 1 in Norway,^37^ 4 in Taiwan,^23^, ^45^, ^65^, ^68^ 2 in the United Kingdom,^20^, ^59^ and 9§ in the United States. Three articles reported data from the CARES (Cardiac Arrest Registry to Enhance Survival) registry, 2 articles from the CAVAS database (Cardiovascular Disease Surveillance), 2 articles from the NHIRD database (National Health Insurance Research), and 3 articles from the UOP (Utstein Osaka Project). Fifteen studies were prospective cohorts, 20 were retrospective cohorts, and 1 was a pilot study for a randomized clinical trial.

The characteristics and quality assessment of included studies are presented in Table S1. The figures for unadjusted analyses are presented in Figure S1. The summary of meta‐analysis results is presented in Table.

**Table 1:** Summary of Meta‐Analysis Results

Outcomes	Studies	Sample size	Effect size, aOR (95% CI)	*P* value	*I* ^2^, %
Survival to discharge, 30 d with good neurological outcome					
Adjusted analyses					
CACs only	5	58 835	1.85 (1.52–2.26)	<0.00001*	75
CACs+high‐volume centers	8	61 733	1.50 (1.18–1.91)	0.0008*	84
CACs+improved‐care centers	11	65 292	2.13 (1.75–2.59)	<0.00001*	73
Unadjusted analyses					
CACs only	7	59 239	2.27 (1.58–3.25)	<0.00001*	90
CACs+high‐volume centers	10	64 512	1.82 (1.35–2.46)	<0.00001*	92
CACs+improved‐care centers	14	64 936	2.16 (1.67–2.81)	<0.00001*	84
Subgroup analyses					
Shockable/nonshockable	5	9129	…	0.006*	…
Prehospital ROSC/no ROSC	5	14 116	…	0.09	…
Survival to discharge, 30 d					
Adjusted analyses					
CACs only	7	25 895	1.92 (1.59–2.32)	<0.00001*	71
CACs+high‐volume centers	9	31 406	1.74 (1.38–2.18)	<0.00001*	85
CACs+improved‐care centers	12	27 762	1.97 (1.71–2.26)	<0.00001*	54
Unadjusted analyses					
CACs only	11	42 323	2.14 (1.75–2.61)	<0.00001*	89
CACs+high‐volume centers	18	84 359	1.98 (1.63–2.40)	<0.00001*	93
CACs+improved‐care centers	19	47 072	2.04 (1.72–2.43)	<0.00001*	84
Subgroup analyses					
Shockable/nonshockable	7	11 207	…	0.73	…
Prehospital ROSC/no ROSC	9	53 592	…	0.005*	…

aOR indicates adjusted odds ratio; CACs, cardiac arrest centers; and ROSC, return of spontaneous circulation.

* *P* < 0.05.

### Survival to 30 Days or Hospital Discharge With Good Neurological Outcome

#### Adjusted Analyses

**Figure 2:**
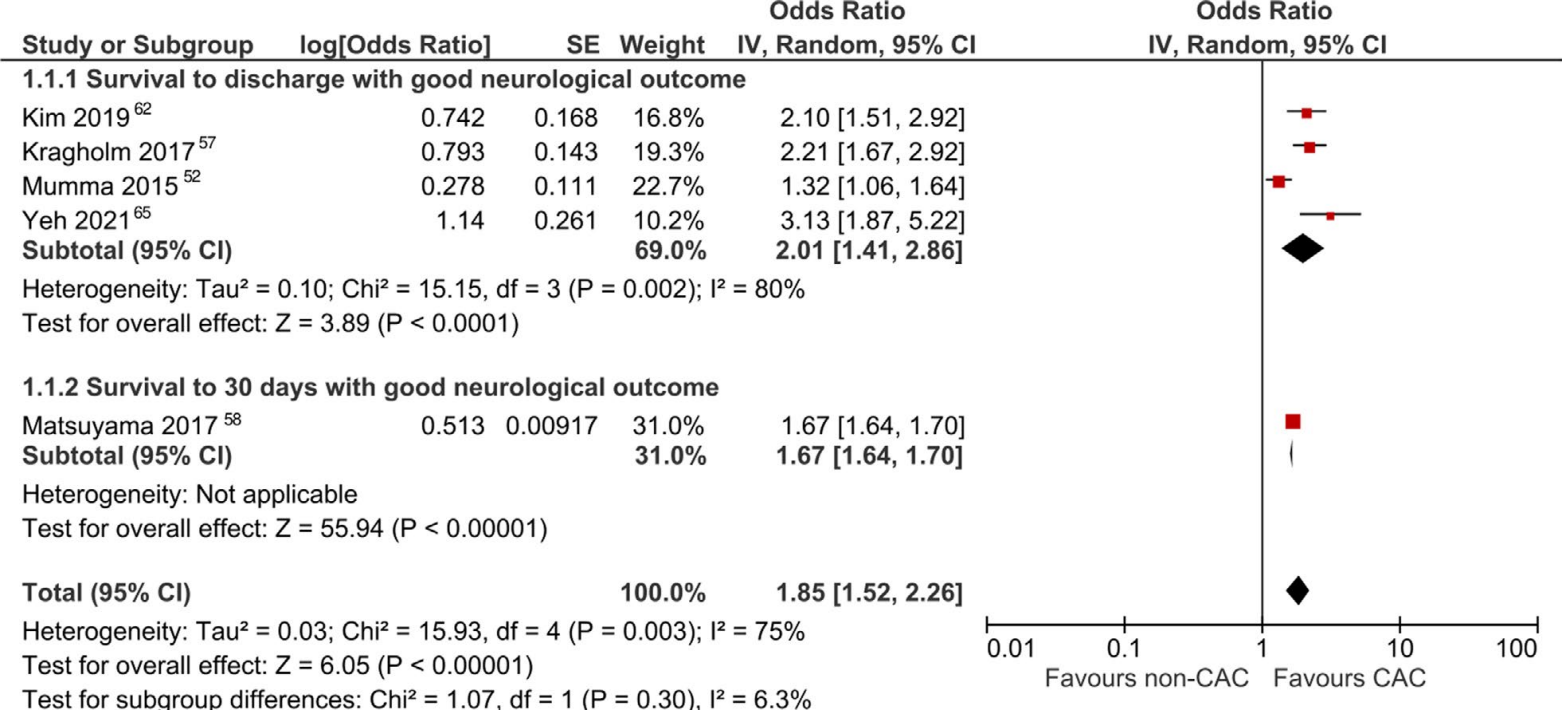
Forest plot for meta‐analysis of adjusted analyses comparing survival with good neurological outcome between cardiac arrest centers (CACs) and non‐CACs using a random‐effects model and the strict definition of CACs. IV indicates inverse variance.

**Figure 3:**
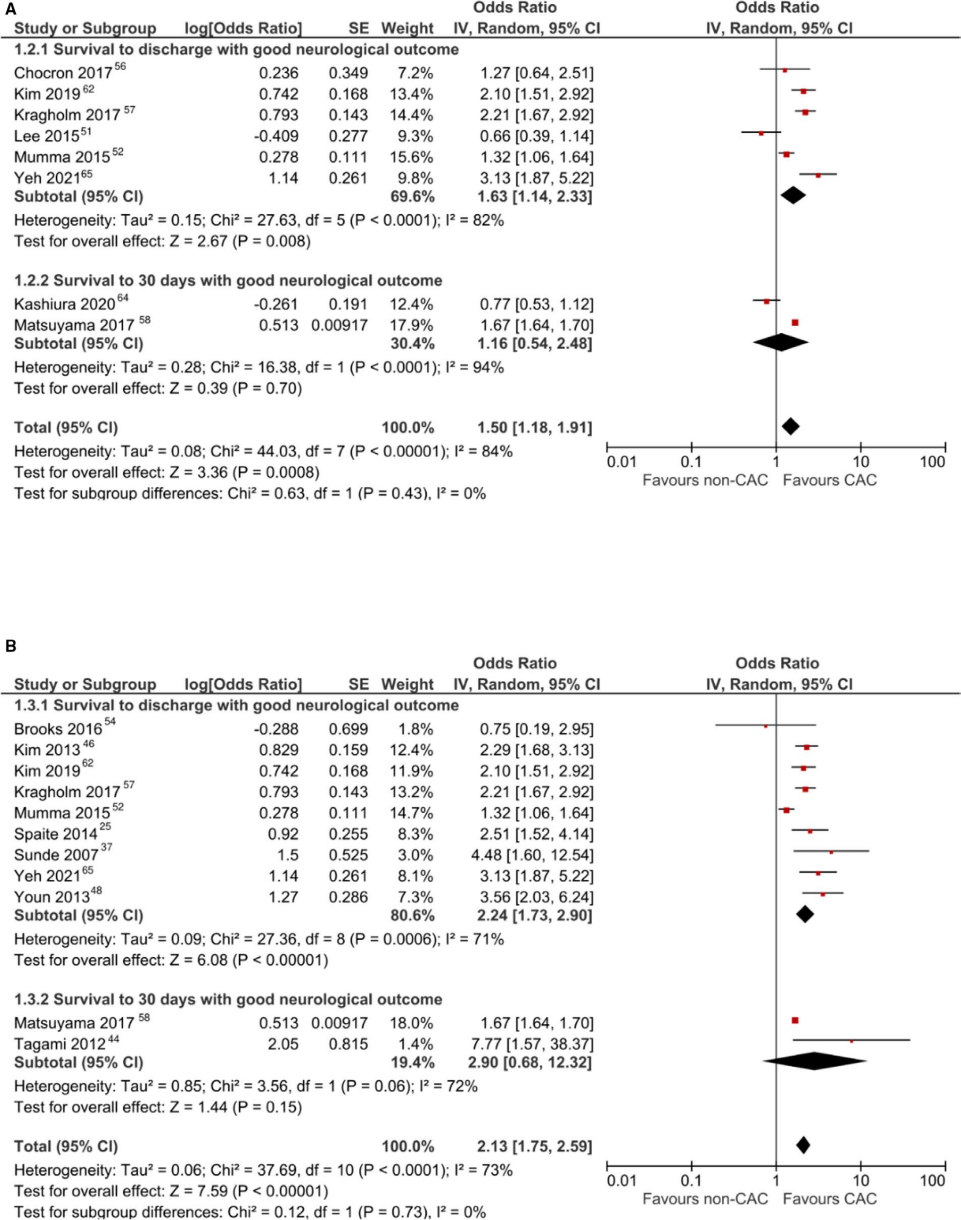
Sensitivity analyses for survival with good neurological outcome using less strict definitions of cardiac arrest centers (CACs). **A**, Forest plot for meta‐analysis of adjusted analyses comparing survival with good neurological outcome between CACs and non‐CACs using a random‐effects model and including high‐volume centers. **B**, Forest plot for meta‐analysis of adjusted analyses comparing survival with good neurological outcome between CACs and non‐CACs using a random‐effects model and including improved‐care centers. IV indicates inverse variance.

Five studies^52^, ^57^, ^58^, ^62^, ^65^ reported aORs for survival to 30 days or hospital discharge with good neurological outcome. Pooled analysis revealed significantly higher survival with good neurological outcome among patients treated at CACs (aOR, 1.85 [95% CI, 1.52–2.26]; Figure 2).^52^, ^57^, ^58^, ^62^, ^65^ There was high between‐study heterogeneity (*I* ^2^, 75%). This result remained significant when using a fixed‐effects model (aOR, 1.67 [95% CI, 1.64–1.70]). On sensitivity analysis, pooled estimates also revealed a significant increase in this outcome among patients treated at CACs when including high‐volume centers (aOR, 1.50 [95% CI, 1.18–1.91]; Figure 3A)^51^, ^52^, ^56^, ^57^, ^58^, ^62^, ^64^, ^65^ and also when including centers with improved postresuscitation care in the definition of CAC (aOR, 2.13 [95% CI, 1.75–2.59]; Figure 3B).‖ When using fixed‐effects models, these results remained significant when including high‐volume centers (aOR, 1.67 [95% CI, 1.64–1.70]) or centers with improved postresuscitation care (aOR, 1.68 [95% CI, 1.65–1.71]). There was no publication bias observed on visual inspection of funnel plots (Figure S2).

#### Unadjusted Analyses

Seven studies^23^, ^53^, ^57^, ^58^, ^59^, ^62^, ^65^ reported ORs for survival to 30 days or hospital discharge with good neurological outcome. Pooled analysis revealed a significantly higher survival with good neurological outcome among patients treated at CACs (OR, 2.27 [95% CI, 1.58–3.25]). Pooled analysis also revealed a significant increase in this outcome among patients treated at CACs when including high‐volume centers (OR, 1.82 [95% CI, 1.35–2.46]) and also when including centers with improved postresuscitation care in the definition of CAC (OR, 2.16 [95% CI, 1.67–2.81]).

#### Subgroup Analysis

**Figure 4:**
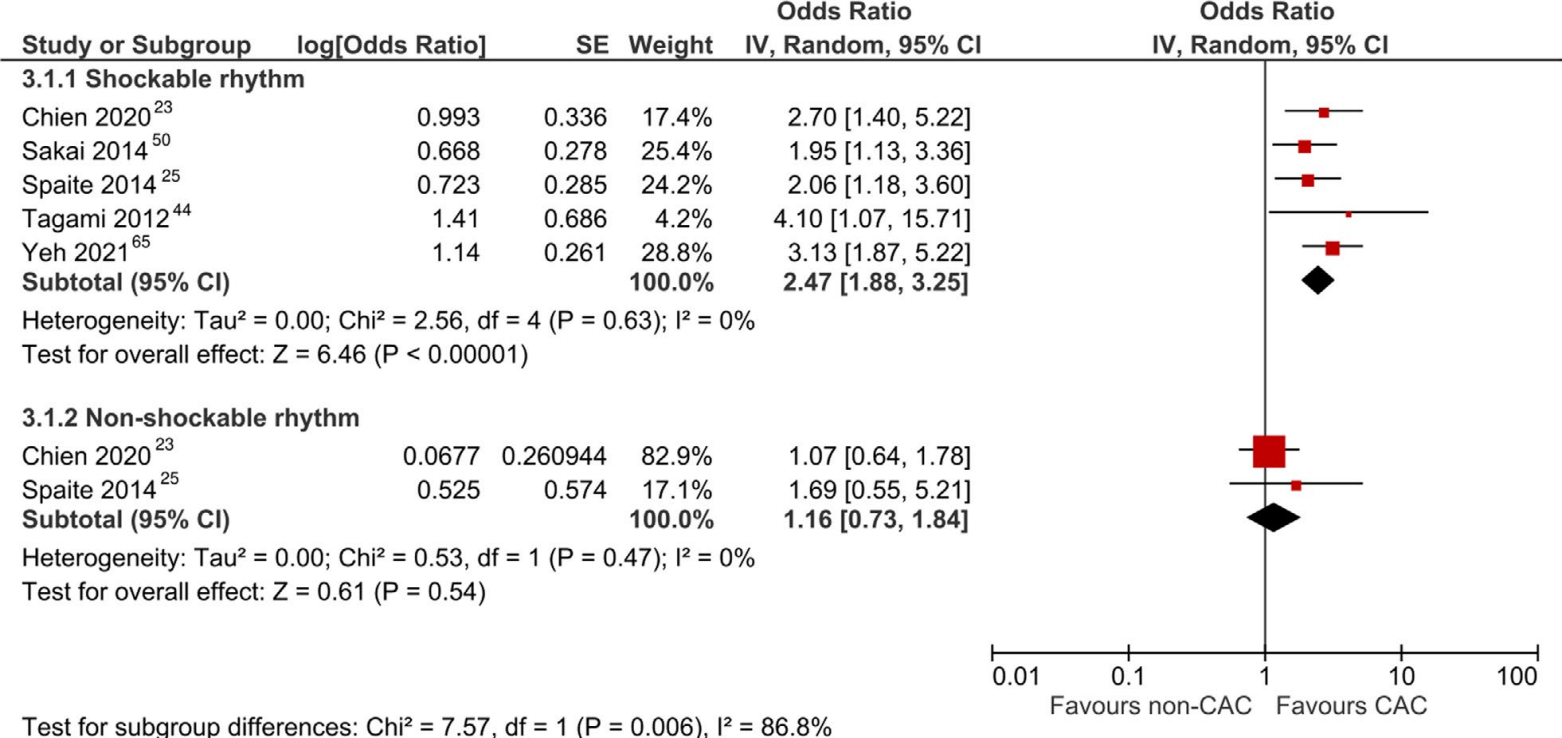
Forest plot for subgroup analysis comparing survival with good neurological outcome between cardiac arrest centers (CACs) and non‐CACs within subgroups of patients with shockable and nonshockable rhythm. IV indicates inverse variance.

Subgroup analysis comparing patients with initial shockable or nonshockable rhythm (Figure 4)^23^, ^25^, ^44^, ^50^, ^65^ revealed a significant increase in survival with good neurological outcome among patients with both shockable (OR, 2.31 [95% CI, 1.77–3.02]) and nonshockable rhythm (OR, 1.16 [95% CI, 0.73–1.84]) when treated at CACs. The treatment effect was significantly greater among patients with initial shockable compared with nonshockable rhythm (*P*=0.006). However, there was no significant difference in survival with good neurological outcome between patients with or without prehospital ROSC (*P*=0.09).

### Survival to 30 Days or Hospital Discharge

#### Adjusted Analyses

**Figure 5:**
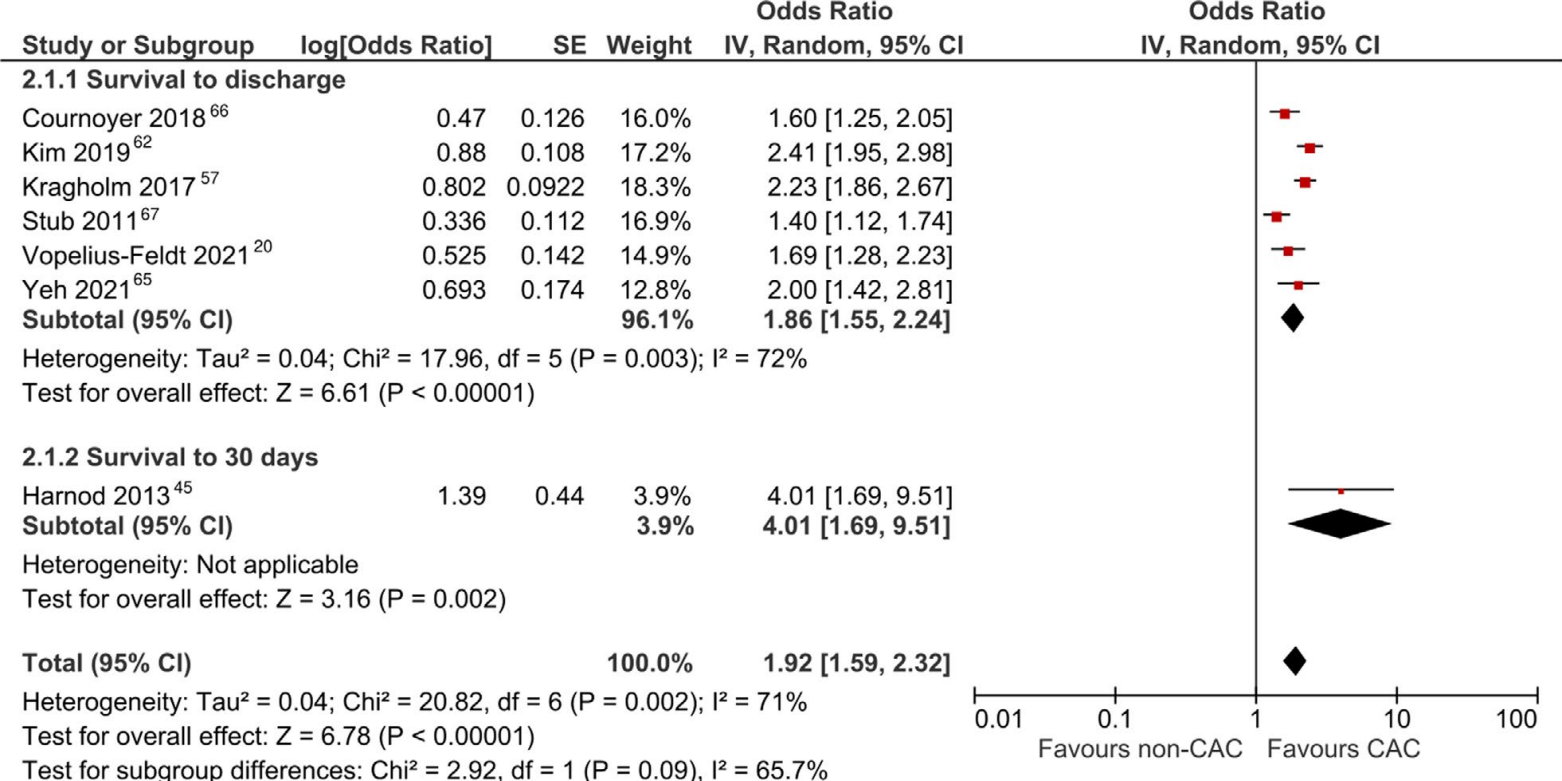
Forest plot for meta‐analysis of adjusted analyses comparing survival between cardiac arrest centers (CACs) and non‐CACs using a random‐effects model and the strict definition of CACs. IV indicates inverse variance.

**Figure 6:**
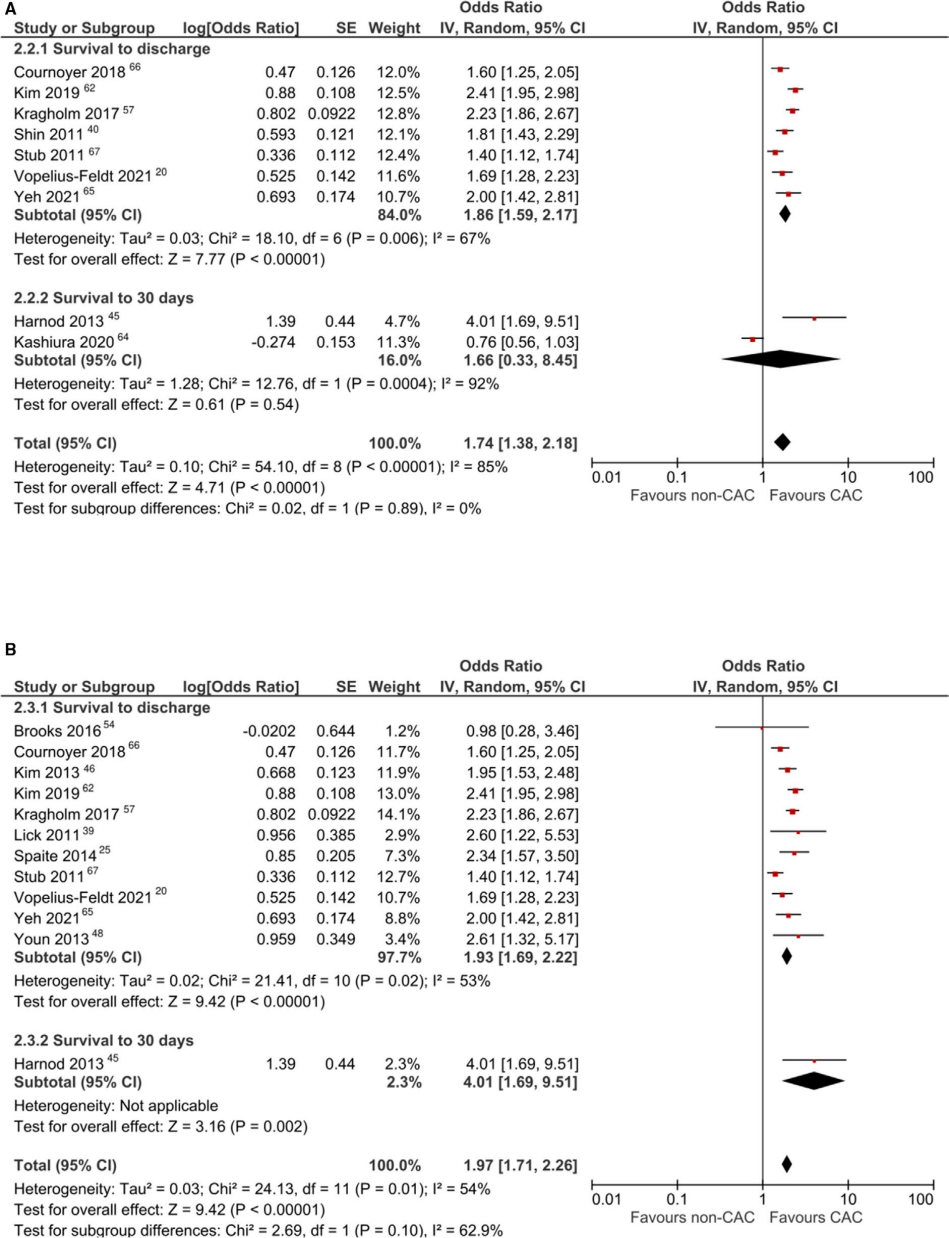
Sensitivity analyses for survival using less strict definitions of cardiac arrest centers (CACs). **A**, Forest plot for meta‐analysis of adjusted analyses comparing survival between CACs and non‐CACs using a random‐effects model and including high‐volume centers. **B**, Forest plot for meta‐analysis of adjusted analyses comparing survival between CACs and non‐CACs using a random‐effects model and including improved‐care centers. IV indicates inverse variance.

Seven studies^20^, ^45^, ^57^, ^62^, ^65^, ^66^, ^67^ reported aORs for survival to 30 days or hospital discharge. Pooled analysis revealed a significant increase in survival among patients treated at CACs (aOR, 1.92 [95% CI, 1.59–2.32]; Figure 5).^20^, ^45^, ^57^, ^62^, ^65^, ^66^, ^67^ There was moderate between‐study heterogeneity (*I* ^2^, 71%). This result remained significant when using a fixed‐effects model (aOR, 1.92 [95% CI, 1.74–2.11]). On sensitivity analysis, pooled estimates also revealed a significant increase in this outcome among patients treated at CACs when including high‐volume centers (aOR, 1.74 [95% CI, 1.38–2.18]; Figure 6A)^20^, ^40^, ^45^, ^57^, ^62^, ^64^, ^65^, ^66^, ^67^ and also when including centers with improved postresuscitation care in the definition of CAC (aOR, 1.97 [95% CI, 1.71–2.26]; Figure 6B).¶ When using fixed‐effects models, these results remained significant when including high‐volume centers (aOR, 1.77 [95% CI, 1.63–1.92]) or centers with improved postresuscitation care (aOR, 1.95 [95% CI, 1.79–2.12]). There was no publication bias observed on visual inspection of funnel plots (Figure S2).

#### Unadjusted Analyses

Eleven studies# reported ORs for survival to 30 days or hospital discharge. Pooled analysis revealed a significant increase in survival among patients treated at CACs (OR, 2.14 [95% CI, 1.75–2.61]). Pooled analysis also revealed a significant increase in this outcome among patients treated at CACs when including high‐volume centers (OR, 1.98 [95% CI, 1.63–2.40]) and also when including centers with improved postresuscitation care in the definition of CAC (OR, 2.04 [95% CI, 1.72–2.43]).

#### Subgroup Analysis

**Figure 7:**
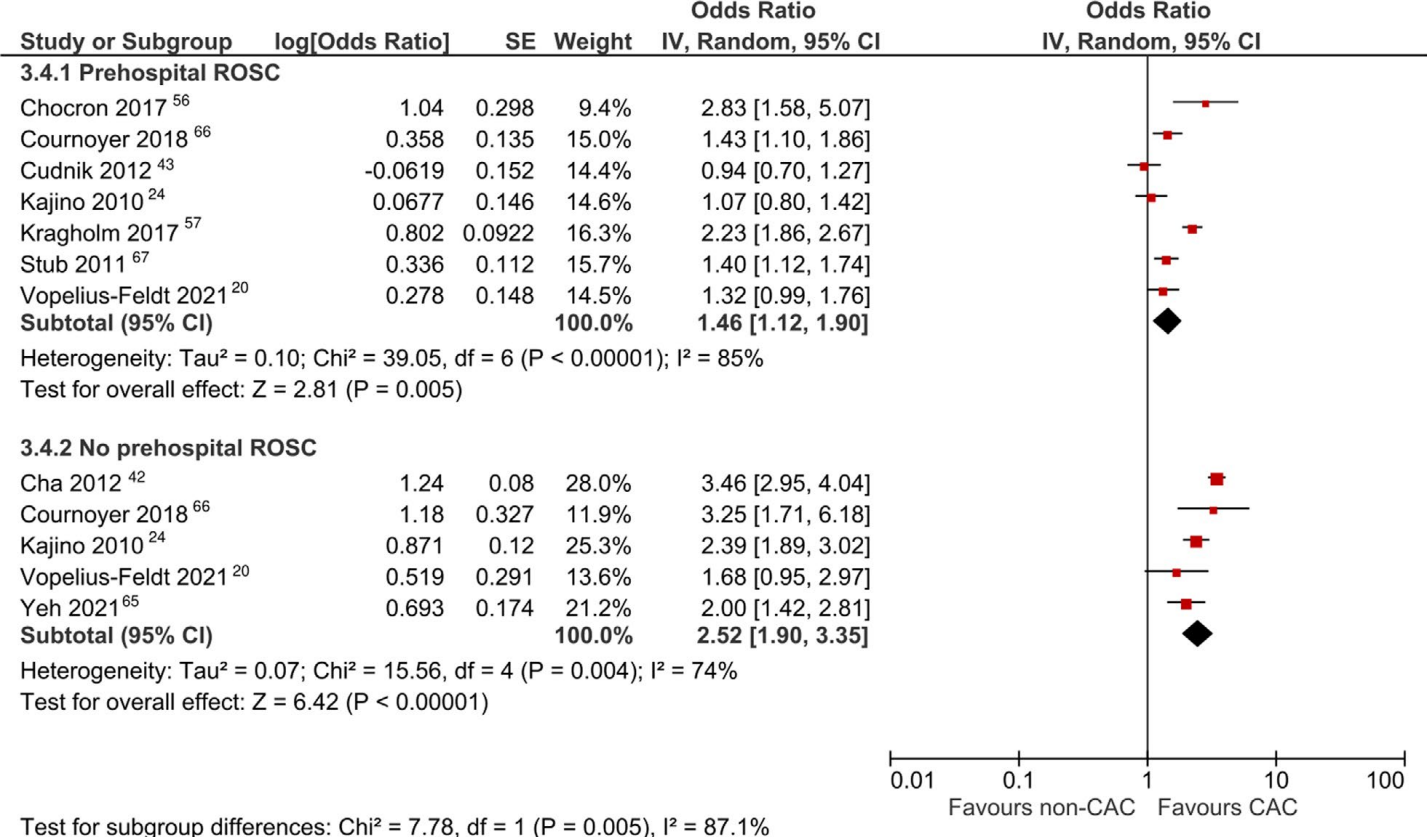
Forest plot for subgroup analysis comparing survival between cardiac arrest centers (CACs) and non‐CACs within subgroups of patients with and without prehospital return of spontaneous circulation (ROSC). IV indicates inverse variance.

Subgroup analysis comparing patients with or without prehospital ROSC (Figure 7)^20^, ^24^, ^42^, ^43^, ^56^, ^57^, ^65^, ^66^, ^67^ revealed a significant increase in survival among patients with prehospital ROSC (OR, 1.46 [95% CI, 1.12–1.90]) as well as among patients without prehospital ROSC (OR, 2.52 [95% CI, 1.90–3.35]). The treatment effect was significantly greater among patients without prehospital ROSC (*P*=0.005). However, there was no significant difference in survival between patients with initial shockable or nonshockable rhythm (*P*=0.73).

### Direct to CAC Versus Transfer to CAC

Only 2 studies^44^, ^49^ reported outcomes for patients directly transported to a CAC versus transferred to a CAC from another hospital. The studies were too heterogeneous to pool, but both reported no significant differences in survival or neurological outcomes between patients directly transported or transferred to a CAC.

## Discussion

The optimal CAC configuration and the benefit of CACs on the survival outcomes of patients with OHCA remain uncertain,^69^ especially for predefined patient subgroups. Only low‐certainty evidence for improved survival in CACs has been demonstrated by previous meta‐analyses (Table S2),^7^, ^10^, ^70^ which were limited by inconsistencies in CAC definitions and the reliance on before‐and‐after study designs vulnerable to inherent biases. This is, to our knowledge, the most up‐to‐date systematic review and meta‐analysis on the topic, with 7 new studies since the last review by Yeung et al, and the first to demonstrate clearly improved survival among patients with OHCA treated in CACs compared with non‐CACs. The results showed (1) significantly improved survival to 30 days or discharge with good neurological outcome and (2) improved survival to 30 days or discharge for patients with OHCA who received care at a CAC (main analysis), regardless of how strictly CACs were defined (sensitivity analyses). Additionally, subgroup analysis suggested that the treatment effect of CACs may be significantly better for patients with shockable rhythm and without prehospital ROSC. On the whole, only 6 studies using before‐and‐after designs were included in this review. These were excluded from the main analysis but included for the sensitivity and subgroup analyses. Taken together, these findings hold implications for the organization of emergency care systems and ambulance diversion strategies for patients with OHCA.

High case volume and aggressive postresuscitation care have been shown to improve outcomes for OHCA, both of which are key features of CACs.^12^, ^61^, ^62^ This analysis demonstrates improved survival and survival with good neurological outcomes both at discharge and 30 days for patients with OHCA treated at CACs, in contrast to a previous meta‐analysis.^10^, ^70^ The quality of included articles using the strict definition of CAC was assessed to be high (≥7), and all were large cohort studies that comprehensively controlled for confounding and did not rely on study designs with historical controls. There was an observed decrease in benefit when including high‐volume centers as CACs as compared with including improved postresuscitation care centers as CACs, possibly suggesting the relatively higher contribution of postresuscitation interventions to the treatment effect, perhaps because cardiac causes of OHCA predominate in this study.^6^, ^65^ However, the consistency of significant benefit across all definitions of CAC indicates that patients should be transported to CACs or even hospitals exhibiting the variable features associated with CACs to improve outcomes.

The 2020 International Liaison Committee on Resuscitation statement also noted that evidence for CACs among subgroups of patients remain inconclusive.^69^ This analysis contributes to this active debate by demonstrating that survival with good neurological outcomes was more pronounced among patients with shockable rhythm and that survival was more pronounced among patients without prehospital ROSC when comparing transport to CACs and non‐CACs. Patients with shockable rhythm have also been associated with OHCA of cardiac causes and may benefit the most from early access to PCI^71^, ^72^ and intensive cardiac care.^65^ Increased benefit in patients without ROSC also partially supports the view favoring quicker transport of patients with refractory OHCA to a hospital^73^, ^74^, ^75^, ^76^ instead of prolonging on‐scene resuscitation,^77^ allowing patients to access advanced critical care and extracorporeal membrane oxygenation. These findings should be interpreted with caution, because the studies included for subgroup analysis were vulnerable to bias but offer preliminary evidence that EMS may consider prioritizing patients with shockable rhythm or without prehospital ROSC for transport to CACs. It should also be considered that patients with nonshockable rhythms inherently have poorer survival and neurological outcomes compared with those with shockable rhythms, which may have contributed to findings of poorer survival and neurological outcomes among such patients regardless of the effect of CACs in relation to non‐CACs. In addition, because patients with nonshockable rhythms still significantly benefited from a CAC albeit to a lesser extent, this analysis does not support depriving these patients from CAC care, but rather, warrants further examination of the associated incremental cost‐effectiveness.

Although transport to a CAC improves outcomes, it remains unclear if EMS should bypass the nearest emergency departments in favor of CACs.^57^, ^78^ It has been suggested that the increase in transport time caused by bypassing the nearest hospital does not substantially affect outcomes after transport to CACs.^23^, ^42^, ^57^, ^76^, ^79^ Other options include initial transport to a non‐CAC with eventual interhospital transfer to a CAC, which seemed to have similar outcomes in this review,^44^, ^49^ but more definitive evidence is required to confirm this finding, in the form of an interventional trial comparing ambulance diversion strategies.

### Strengths and Limitations

This is the largest systematic review and meta‐analysis of evidence for the benefits of CACs conducted to date, involving OHCA registries and databases from various nations and a sample size of 147 943 patients. However, differences across geographical regions may have led to the high statistical heterogeneity encountered in various analyses. Outcomes at both 30 days and at discharge were pooled, also contributing to heterogeneity, because time points for discharge may differ based on health system. However, we found that our results remained consistent both on pooling survival at 30 days with survival at discharge, and also when examining each of these separately. Another limitation is that the included studies used largely similar but not identical covariates for the adjustment of ORs, which may lead to residual confounding. As a whole, the interpretation of our results should also consider that specific levels of care at non‐CAC hospitals were inconsistently defined.

Subgroup analyses should be interpreted carefully given existing selection bias by EMS and smaller sample sizes. The conclusion that patients with shockable rhythms do better when transported to CACs may have been driven by a higher proportion of ST‐segment–elevation myocardial infarction within this group, hence accounting for better outcomes thanks to the presence of cardiac catheterization laboratories in CACs. Furthermore, observational studies are inherently susceptible to selection and observation biases. High‐quality randomized clinical trials are therefore urgently needed to confirm present findings. Evidence for direct transport or transfer to a CAC was inconclusively assessed. Non‐English language articles were also excluded.

## Conclusions

CACs improved survival and neurological outcomes at discharge or 30 days among patients with OHCA, regardless of how CACs were defined. There was preliminary evidence for EMS to consider transport to CACs, especially for patients with shockable rhythm or patients without prehospital ROSC. High‐quality data are needed to confirm these findings and conclusively assess whether patients should bypass the nearest hospital to be transported to a CAC versus transferred to a CAC from the nearest hospital.

## Appendix

The National TTM Workgroup, which is part of the Unit for Prehospital Emergency Care, Ministry of Health, Singapore, currently consists of: Shiang‐Hu Ang, Ruth Weixian Chen, Yew Woon Chia (chairperson), Enoch Hin Kei Chan, Ee Ling Goh, Andrew Fu Wah Ho, Vui Kian Ho, Hong Khai Lau, Eng Kiang Lee, Benjamin Sieu‐Hon Leong, Jia Hao Lim, Shir Lynn Lim, Julian Kenrick Xingyuan Loh, Jimmy Heng Ann Ong, Marcus Eng Hock Ong, Kah Hua Peck, Daniel Yong Jing Quek, Christopher Ying Hao Seet, Shobbit Swarup, and Thon Hon Yong.

## Sources of Funding

Dr Ho was supported by the Estate of Tan Sri Khoo Teck Puat (Khoo Clinical Scholars Programme), Khoo Pilot Award (KP/2019/0034), Duke‐NUS Medical School, and National Medical Research Council (NMRC/CS_Seedfd/012/2018). This study was conducted as part of the National TTM Workgroup, a unit under the Unit for Prehospital Emergency Care, Ministry of Health, Singapore.

## Disclosures

Dr Ong reports funding from the Zoll Medical Corporation for a study involving mechanical cardiopulmonary resuscitation devices and an advisory relationship with Global Healthcare SG, a commercial entity that manufactures cooling devices. The remaining authors have no disclosures to report.

## Supporting information

Data S1
Tables S1–S4
Figures S1–S2

## Footnotes

^§^
References 25, 38, 39, 41, 43, 52, 54, 57, 61.

^‖^
References 25, 37, 44, 46, 48, 52, 54, 57, 58, 62, 65.

^¶^
References 20, 25, 39, 45, 46, 48, 54, 57, 62, 65, 66, 67.

^#^
References 20, 24, 47, 53, 57, 59, 62, 65, 66, 67, 68.
