# Supplementary material for: Impact of Cardiac Arrest Centers on the Survival of Patients With Nontraumatic Out‐of‐Hospital Cardiac Arrest: A Systematic Review and Meta‐Analysis
Source: J Am Heart Assoc. 2021 Dec 20;11(1):e023806. doi: 10.1161/JAHA.121.023806 (PMC9075197; doi:10.1161/JAHA.121.023806)
Supplement: Supplementary file 1 — Data S1 Tables S1–S4 Figures S1–S2 [file JAH3-11-e023806-s001.pdf]

SUPPLEMENTAL MATERIAL

Data S1. Detailed Search Strategy.

Search strategy for Medline

1: Cardiac Care Facilities/
2: Cardiology Service, Hospital/
3: Regional Medical Programs/
4: ((heart or cardi*) adj3 (attack or arrest) adj3 (centre* or center*)).ab,kf,ti.
5: (cardiac resuscitation center* or cardiac resuscitation centre* or regional cardiac resuscitation).ab,kf,ti.
6: (regional system* or network or hospital volume or patient volume).ab,kf,ti.
7: (Cardiac Receiving Center* or Cardiac Receiving Centre*).ab,kf,ti.
8: “Cardiac Care Facilit*”.ab,kf,ti.
9: (Cardi* adj2 (Centre* or Center*)).ab,kf,ti.
10: (Cardiology adj1 (Service or care) adj2 Hospital).ab,kf,ti.
11: cardiac catheterisation laboratory.ab,kf,ti.
12: (CAC or CACs).ab,kf,ti.
13: ((post cardiac arrest or postcardiac arrest) adj1 (care or treatment)).ab,kf,ti.
14: ((post resuscitation or postresuscitation) adj1 (care or treatment)).ab,kf,ti.
15: fifth link.ab,kf,ti.
16: Tertiary Care Centers/
17: (Tertiary adj1 (care or Center* or Centre*)).ab,kf,ti.
18: Cardiac Arrest Registry.ab,kf,ti.
19: (“Critical care medical center*” or “Critical care medical centre*”).ab,kf,ti.
20: (“critical care centre*” or “critical care center*”).ab,kf,ti.
21: or/1-20
22: exp heart arrest/
23: cardiopulmonary resuscitation/ or advanced cardiac life support/
24: Out of Hospital Cardiac Arrest.ab,kf,ti.
25: OHCA.ab,kf,ti.
26: return of spontaneous circulation.ab,kf,ti.
27: ROSC.ab,kf,ti.
28: ((heart or cardiac or cardiovascular) adj1 arrest).ab,kf,ti.
29: asystole.ab,kf,ti.
30: pulseless electrical activity.ab,kf,ti.
31: Advanced Cardiac Life Support.ab,kf,ti.
32: ACLS.ab,kf,ti.

33: Ventricular Fibrillation/
34: (cardiopulmonary arrest or cardiopulmonary resuscitation).ab,kf,ti.
35: (cardio-pulmonary arrest or cardio-pulmonary resuscitation or CPR) .ab,kf,ti.
36: code blue.ab,kf,ti.
37: or/22-36
38: and/21,37
39: exp Organ Transplantation/ or “transplant”.ab,kf,ti.
40: 38 not 39
41: Animals/ not (Animals/ and Humans/)
42: 40 not 41
43: (exp Pediatrics/ or exp CHILD/) not exp Adult/
44: 42 not 43
45: (letter or comment or editorial or note or news).pt.
46: 44 not 45
47: Case Reports/ or (case report or case series).ti.
48: 46 not 47
49: remove duplicates from 48

Search strategy for Embase

1: ‘heart center’/de
2: ‘cardiology service’/de
3: ‘regional medical program*’:ab,ti,kw
4: ((heart or cardi*) NEAR/3 (attack OR arrest) NEAR/3 (centre* or center*)):ab,ti,kw
5: ‘cardiology service*’:ab,ti,kw
6: ‘cardiac resuscitation center*’:ab,ti,kw OR ‘cardiac resuscitation centre*’:ab,ti,kw OR ‘regional cardiac resuscitation’:ab,ti,kw
7: ‘regional system*’:ab,ti,kw OR ‘network’:ab,ti,kw OR ‘hospital volume’:ab,ti,kw OR ‘patient volume’:ab,ti,kw
8: ‘cardiac receiving center*’:ab,ti,kw OR ‘cardiac receiving centre*’:ab,ti,kw
9: ‘cardiac care facilit*’:ab,ti,kw
10: (cardi* NEAR/2 (centre* or center*)):ab,ti,kw
11: (cardiology NEAR/1 (service OR care) NEAR/2 hospital):ab,ti,kw
12: cardiac AND catheterisation AND laboratory:ab,ti,kw
13: ‘cardiac catheterisation laboratory’:ab,ti,kw
14: cac:ab,ti,kw OR cacs:ab,ti,kw
15: ((‘post cardiac arrest’ OR ‘postcardiac arrest’) NEAR/1 (care OR treatment)):ab,ti,kw
16: ((‘post resuscitation’ OR ‘postresuscitation’) NEAR/1 (care OR treatment)):ab,ti,kw
17: ‘fifth link’:ab,ti,kw

18: ‘tertiary care center’/de
19: (tertiary NEAR/1 (care OR center* OR centre*)):ab,ti,kw
20: ‘cardiac arrest registry’:ab,ti,kw
21: ‘critical care medical center*’:ab,ti,kw OR ‘critical care medical centre*’:ab,ti,kw
22: ‘critical care centre*’:ab,ti,kw OR ‘critical care center*’:ab,ti,kw
23: #1 OR #2 OR #3 OR #4 OR #5 OR #6 OR #7 OR #8 OR #9 OR #10 OR #11 OR #12 OR #13 OR #14 OR #15 OR #16 OR #17 OR #18 OR #19 OR #20 OR #21 OR #22
24: ‘heart arrest’/exp
25: ‘cardiac life support’:ab,ti,kw
26: ‘ohca’:ab,ti,kw
27: ‘return of spontaneous circulation’/de
28: ((heart OR cardiac OR cardiovascular) NEAR/1 arrest):ab,ti,kw
29: asystole:ab,ti,kw
30: ‘pulseless electrical activity’:ab,ti,kw
31: acls:ab,ti,kw
32: ‘heart ventricle fibrillation’/de
33: ‘cardiopulmonary arrest’:ab,ti,kw OR ‘cardiopulmonary resuscitation’:ab,ti,kw
34: ‘cardio-pulmonary arrest’:ab,ti,kw OR ‘cardio-pulmonary resuscitation’:ab,ti,kw OR ‘cpr’:ab,ti,kw
35: ‘code blue’:ab,ti,kw
36: #24 OR #25 OR #26 OR #27 OR #28 OR #29 OR #30 OR #31 OR #32 OR #33 OR #34 OR #35
37: #23 AND #36
38: ‘organ transplantation’/exp OR ‘transplant’:ab,ti,kw
39: #37 NOT #38
40: ‘animal’/exp NOT (‘animal’/exp AND ‘human’/exp)
41: #39 NOT #40
42: (‘pediatrics’/exp OR ‘child’/exp) NOT ‘adult’/de
43: #41 NOT #42
44: ‘article’/it
45: #43 AND #44
46: ‘case report’/de OR ‘case study’/de OR ‘case study’:ti OR ‘case series’:ti
47: #45 NOT #46

Search strategy for Cochrane CENTRAL

1: MeSH descriptor: [Cardiac Care Facilities] this term only
2: MeSH descriptor: [Cardiology Service, Hospital] this term only
3: MeSH descriptor: [Regional Medical Programs] this term only
4: ((heart or cardi*) NEAR/3 (attack or arrest) NEAR/3 (centre* or center*)):ti,ab,kw

5: (cardiac resuscitation center* or cardiac resuscitation centre* or regional cardiac resuscitation):ti,ab,kw
6: (regional system* or network or hospital volume or patient volume):ti,ab,kw
7: (Cardiac Receiving Center* or Cardiac Receiving Centre*):ti,ab,kw
8: ("Cardiac Care Facility" or "Cardiac Care Facilities"):ti,ab,kw
9: (Cardi* NEAR/2 (Centre* or Center*)):ti,ab,kw
10: (Cardiology NEAR/1 (Service or Care) NEAR/2 Hospital):ti,ab,kw
11: (cardiac catheterisation laboratory):ti,ab,kw
12: (CAC or CACs):ti,ab,kw
13: (("post cardiac arrest" or "postcardiac arrest") NEAR/1 (care or treatment)):ti,ab,kw
14: (("post resuscitation" or "postresusciation") NEAR/1 (care or treatment)):ti,ab,kw
15: (fifth link):ti,ab,kw
16: MeSH descriptor: [Tertiary Care Centers] this term only
17: (Tertiary NEAR/1 (Care or Center* or Centre*)):ti,ab,kw
18: (Cardiac Arrest Registry):ti,ab,kw
19: ("Critical care medical center*" or "Critical care medical centre*"):ti,ab,kw
20: ("critical care center*" or "critical care centre*"):ti,ab,kw
21: #1 or #2 or #3 or #4 or #5 or #6 or #7 or #8 or #9 or #10 or #11 or #12 or #13 or #14 or #15 or #16 or #17 or #18 or #19 or #20
22: MeSH descriptor: [Heart Arrest] explode all trees
23: MeSH descriptor: [Cardiopulmonary Resuscitation] this term only
24: MeSH descriptor: [Advanced Cardiac Life Support] this term only
25: (Out of Hospital Cardiac Arrest):ti,ab,kw
26: OHCA:ti,ab,kw
27: (return of spontaneous circulation):ti,ab,kw
28: ROSC:ti,ab,kw
29: ((heart or cardiac or cardiovascular) NEAR/1 arrest):ti,ab,kw
30: asystole:ti,ab,kw
31: (pulseless electrical activity):ti,ab,kw
32: (Advanced Cardiac Life Support):ti,ab,kw
33: ACLS:ti,ab,kw
34: MeSH descriptor: [Ventricular Fibrillation] this term only
35: (cardiopulmonary arrest or cardiopulmonary resuscitation):ti,ab,kw
36: (cardio-pulmonary arrest or cardio-pulmonary resuscitation or CPR):ti,ab,kw
37: (code blue):ti,ab,kw
38: #22 or #23 or #24 or #25 or #26 or #27 or #28 or #29 or #30 or #31 or #32 or #33 or #34 or #35 or #36 or #37
39: #21 and #38
40: MeSH descriptor: [Organ Transplantation] explode all trees
41: #40 or transplant:ti,ab,kw
42: #39 not #41
43: MeSH descriptor: [Animals] this term only

44: MeSH descriptor: [Humans] this term only
45: #43 not (#43 and #44)
46: #42 not #45
47: MeSH descriptor: [Pediatrics] explode all trees
48: MeSH descriptor: [Child] explode all trees
49: MeSH descriptor: [Adult] explode all trees
50: (#47 or #48) not #49
51: #46 not #50
52: (article):pt
53: #51 and #52
54: MeSH descriptor: [Case Reports] this term only
55: #54 or (case report):ti or (case series):ti
56: #53 not #55

Table S1. Summary of Included Studies

Author	Country	Study Design	Comparison	Sample size	Age (mean SD)	Male	CAC description	NOS
Balian 2019	USA	Retrospective cohort	High volume centres	613	65 +/- 23	354	High volume center (84-205 cases/5 years)	7
			Low volume centres	577	64 +/- 22	371		
Brooks 2016	USA	Prospective cohort	Improved care centres	151	64.7 +/- 16	117	Post-arrest consult team: PCI, TTM, NP	7
			Usual care	855	65.3 +/- 16.6	567		
Cha 2012	South Korea	Retrospective cohort	High volume centres	11777	65 (51-75)*	7742	High volume center (>33 cases/year)	7
			Low volume centres	15885	66 (53-76)*	10438		
Chien 2020	Taiwan	Prospective cohort	CACs	2578	69.4 +/- 16.9	1782	CAC: 24/7 PCI, TTM, ECMO	7
			Non-CACs	2578	69.3 +/- 17	1757		
Chocron 2017	France	Retrospective cohort	High volume centres	917	60.1 +/- 15.4	658	High volume center (>/=1000 ICU/year, >/= 15 cases/ year): 24/7 PCI, TTM, CAG	7
			Low volume centres	91	66.6 +/- 16.6	56		
Cournoyer 2018	Canada	Retrospective cohort	CACs	2389	67.1 +/- 16.4	1629	PCI-capable STEMI center: 24/7 PCI, CAG	7
			Non-CACs	2533	67.4 +/- 16.8	1608		
Cudnik 2012	USA			928	62.6	566		7

		Prospective cohort	High volume centres<br>Low volume centres	697	63.7	397	High volume center (>40 cases/year): 24/7 PCI, TTM	
Gaieski 2009	USA	Prospective cohort	Improved care centres	18	57 (20-86)*	12	Post-resuscitation algorithm: TTM, PCI, EGDHO	7
			Usual care	18	67 (35-87)*	9		
Harnod 2013	Taiwan	Retrospective cohort	CACs	435			Critical care medical center	9
			Non-CACs	457				
Kajino 2010	Japan	Prospective cohort	CACs	2881		1781	Critical care medical center	8
			Non-CACs	7502		4179		
Kang 2014	South Korea	Retrospective cohort	Transferred to CAC	41	54.6 +/- 17.6	27	CAC: PCI, CAG, TTM	8
			Direct transport to CAC	50	48.9 +/- 16.9	28		
Kashiura 2020	Japan	Retrospective cohort	High volume centres	912	71 (60-81)*	577	High volume centre (79-118 cases/15 months)	8
			Low volume centres	889	71 (60-82)*	575		
Kim 2013	South Korea	Retrospective cohort	Improved care centres	678	57.6 +/- 14.7	520	Active post-resuscitation care: TTM, PCI, CABG	8
			Usual care	678	57.1 +/- 15.7	511		
Kim 2019	South Korea	Retrospective cohort	CACs	4036	68 (54-79)*	2632	Cardiac resuscitation centre: 24/7 PCI, TTM	7
			Non-CACs	5876	72 (57-81)*	3654		
Kragholm 2017	USA	Prospective cohort	CACs	1359	65 (55-75)*	827	PCI center: 24/7 PCI, TTM	7
			Non-CACs	148	67 (56-78)*	83		
Lai 2018	Taiwan		CACs	2255			Critical care medical center	9

		Retrospective cohort	Non-CACs	2353				
Lee 2015	South Korea	Retrospective cohort	High volume centres	289	58 (47-70)*	201	High volume center (>15.5 cases/year): TTM	7
			Low volume centres	289	58 (48-69.5)*	200		
Lick 2011	USA	Prospective cohort	Improved care centres	247	62 +/- 15.6	173	CAC: PCI, TTM, ICD	7
			Usual care	106	68 +/- 14.6	75		
Matsuyama 2017	Japan	Retrospective cohort	CACs	15118			Critical care medical center: 24/7 PCI, ECMO	8
			Non-CACs	24847				
Mumma 2015	USA	Retrospective cohort	CACs	3340	65 (53-77)*	1956	STEMI centers: 24/7 PCI, TTM	7
			Non-CACs	2523	68 (55-79)*	1379		
Park 2019	South Korea	Retrospective cohort	High volume centres	1200		804	High volume center (>100 OHCA cases/ year): PCI, TTM, ECMO	7
			Low volume centres	2608		1760		
Patterson 2017	UK	Randomised controlled trial	CACs	18			CAC: 24/7 PCI, TTM, CAG	Some concerns†
			Non-CACs	15				
Sakai 2014	Japan	Prospective cohort	CACs	112		91	Critical care medical center: PCI, TTM, ECMO	7
			Non-CACs	140				
Schober 2016	Austria	Prospective cohort	High volume centres	378	60 (49-70)*	276	High volume center (>100 OHCA cases/ year): 24/7 CAG, TTM	8
			Low volume centres	269	66 (52-75)*	181		
Seiner 2018				61		46		6

	Czech Republic	Prospective cohort	Improved care centres<br>Usual care	147		117	After designation as CAC, post-cardiac arrest treatment: PCI, TTM, MV, ICD	
Shin 2011	South Korea	Retrospective cohort	High volume centres	3533	60.9 +/- 19.4	2270	High volume center (>68 OHCA cases/ 2 years)	7
			Low volume centres	3533	60.5 +/- 18.6	2322		
Soholm 2013	Denmark	Retrospective cohort	CACs	761		581	Tertiary centre: PCI, TTM, CAG	8
			Non-CACs	457	68 +/- 14	278		
Soholm 2015	Denmark	Prospective cohort	CACs	586	63 +/- 15	433	Tertiary centre: PCI, TTM, ICD, EGDHO	6
			Non-CACs	492	68 +/- 14	303		
Spaite 2014	USA	Prospective cohort	Improved care centres	1737	63 (62.2-63.8)*	1132	After receiving designation as cardiac receiving center: PCI, CAG, TTM	7
			Usual care	440	63.9 (62.4-65.4)*	280		
Stub 2011	Australia	Retrospective cohort	CACs	1816		1294	Cardiac centre: 24/7 PCI and interventional cardiac services	7
			Non-CACs	890		571		
Sunde 2007	Norway	Prospective cohort	Improved care centres	61	63 +/- 14	50	Standardised treatment protocol: PCI, TTM, MV, EGDHO	7
			Usual care	58	68 +/- 12	46		
Tagami 2012	Japan	Prospective cohort	Improved care centres	712	76.3 +/- 13.9	397	Post-resuscitation care bundle: TTM, CAG, ECMO	8
			Usual care	770	75.3 +/- 14.5	458		
	UK		CACs	2184	72 (60-82)*	1374	24/7 PCI centres: TTM	7

Vopelius-Feldt 2021		Retrospective cohort	Non-CACs	2184	73 (62-82)*	1430		
Walters 2011	USA	Prospective cohort	Improved care centres	29	62 +/- 10	19	Care bundle: PCI, TTM, EGDHO	7
			Usual care	26	64 +/- 15	18		
Yeh 2021	Taiwan	Retrospective cohort	CACs	1222	62.61 +/- 15.67	926	Heart centre: 24/7 PCI, TTM	7
			Non-CACs	366	61.95 +/- 16.19	288		
Youn 2013	South Korea	Retrospective cohort	Improved care centres	168	62.3 +/- 19.3	111	Post-cardiac arrest care package: PCI, TTM, MV, EGDHO, NP	7
			Usual care	149	65 +/- 14.4	106		

CAC: Cardiac arrest centre; NOS: Newcastle-Ottawa score; STEMI: ST-elevation myocardial infarction; PCI: percutaneous coronary intervention; CAG: Coronary artery angiography; TTM: targeted temperature management; ECMO: extracorporeal membrane oxygenation, MV: mechanical ventilation, NP: neuroprognostication, EGDHO: early goal-directed hemodynamic optimization, ICD: implantable cardioverter defibrillator
*Median, IQR, †Cochrane Risk of Bias 2 Tool

Table S2. Summary of Pre-existing Meta-Analyses

Author	Year	Comparison	Definition of CAC	Findings
Yeung et al	2019	CAC vs non-CAC	Accepted 'cardiac arrest centre' or 'regionalized cardiac arrest care' or 'high case volume centres' of similar description in the literature.	Very low certainty evidence suggests that post-cardiac arrest care at cardiac arrest centres is associated with improved survival with favourable neurological outcome at hospital discharge and improved survival to hospital discharge. Care at CACs did not improve survival to 30 days with favourable neurological outcome and survival to 30 days. There remains a need of high quality data individual patient data meta-analysis and or data from randomised trials to fully elucidate the impact of CAC.
Lipe et al	2018	CAC vs non-CAC	To be considered a cardiac resuscitation center, a hospital was required to have both PCI capability and TTM capability as defined by the American Heart Association.	Adult patients suffering from an OHCA transported to cardiac resuscitation centers seem to have better outcomes than their counterparts. It is reasonable to transport these patients directly to cardiac resuscitation centers (class IIa, level of evidence B-nonrandomized). Future studies should further clarify how long a bypass time is tolerable for these patients, especially for the subpopulation of patients not having experienced prehospital ROSC.
Storm et al	2019	Structured pathways of care vs usual care	Studies implemented a structured care pathway, defined as an organized treatment protocol which was determined a priori, implemented postcardiac arrest and during the acute hospitalization, and had more than one intervention (e.g., PCI and TTM).	Our findings support a highly organized approach to postcardiac arrest care, in which a cluster of evidence-based interventions are delivered by a specialized interdisciplinary team. Given the overall low certainty of evidence, however, definitive recommendations will depend on confirmation in additional high-quality studies. Additionally, results presented here provide the rationale for future studies which will test optimal combinations and timing of interventions, and which will integrate a structured approach to neurologic prognostication with the goal of further ameliorating care and outcomes in this population. There is also a need for new research which accounts for heterogeneity inherent in this population and validates personalized approaches based on biological subtypes.

CAC: Cardiac arrest centers; PCI: Percutaneous coronary intervention; TTM: Targeted temperature management

Table S3. PRISMA 2020 Checklist

Section and Topic	Item #	Checklist item	Location where item is reported
**TITLE**			
Title	1	Identify the report as a systematic review.	Page 1
**ABSTRACT**			
Abstract	2	See the PRISMA 2020 for Abstracts checklist.	Page 3
**INTRODUCTION**			
Rationale	3	Describe the rationale for the review in the context of existing knowledge.	Page 8-9
Objectives	4	Provide an explicit statement of the objective(s) or question(s) the review addresses.	Page 9
**METHODS**			
Eligibility criteria	5	Specify the inclusion and exclusion criteria for the review and how studies were grouped for the syntheses.	Page 11
Information sources	6	Specify all databases, registers, websites, organisations, reference lists and other sources searched or consulted to identify studies. Specify the date when each source was last searched or consulted.	Page 10
Search strategy	7	Present the full search strategies for all databases, registers and websites, including any filters and limits used.	Page 10; Supp Material 1
Selection process	8	Specify the methods used to decide whether a study met the inclusion criteria of the review, including how many reviewers screened each record and each report retrieved, whether they worked independently, and if applicable, details of automation tools used in the process.	Page 11
Data collection process	9	Specify the methods used to collect data from reports, including how many reviewers collected data from each report, whether they worked independently, any processes for obtaining or confirming data from study investigators, and if applicable, details of automation tools used in the process.	Page 12-14
Data items	10a	List and define all outcomes for which data were sought. Specify whether all results that were compatible with each outcome domain in each study were sought (e.g. for all measures, time points, analyses), and if not, the methods used to decide which results to collect.	Page 12
	10b	List and define all other variables for which data were sought (e.g. participant and intervention characteristics, funding sources). Describe any assumptions made about any missing or unclear information.	Page 12; Table 1
Study risk of bias assessment	11	Specify the methods used to assess risk of bias in the included studies, including details of the tool(s) used, how many reviewers assessed each study and whether they worked independently, and if applicable, details of automation tools used in the process.	Page 13-14
Effect measures	12	Specify for each outcome the effect measure(s) (e.g. risk ratio, mean difference) used in the synthesis or presentation of results.	Page 12-13
Synthesis methods	13a	Describe the processes used to decide which studies were eligible for each synthesis (e.g. tabulating the study intervention characteristics and comparing against the planned groups for each synthesis (item #5)).	Page 11-12
	13b	Describe any methods required to prepare the data for presentation or synthesis, such as handling of missing summary statistics, or data	Page 12-13

Section and Topic	Item #	Checklist item	Location where item is reported
		conversions.	
	13c	Describe any methods used to tabulate or visually display results of individual studies and syntheses.	Page 13
	13d	Describe any methods used to synthesize results and provide a rationale for the choice(s). If meta-analysis was performed, describe the model(s), method(s) to identify the presence and extent of statistical heterogeneity, and software package(s) used.	Page 12-13
	13e	Describe any methods used to explore possible causes of heterogeneity among study results (e.g. subgroup analysis, meta-regression).	Page 13
	13f	Describe any sensitivity analyses conducted to assess robustness of the synthesized results.	Page 13
Reporting bias assessment	14	Describe any methods used to assess risk of bias due to missing results in a synthesis (arising from reporting biases).	Page 13
Certainty assessment	15	Describe any methods used to assess certainty (or confidence) in the body of evidence for an outcome.	Page 13-14
**RESULTS**			
Study selection	16a	Describe the results of the search and selection process, from the number of records identified in the search to the number of studies included in the review, ideally using a flow diagram.	Figure 1
	16b	Cite studies that might appear to meet the inclusion criteria, but which were excluded, and explain why they were excluded.	Figure 1
Study characteristics	17	Cite each included study and present its characteristics.	Supp Material 2
Risk of bias in studies	18	Present assessments of risk of bias for each included study.	Supp Material 2
Results of individual studies	19	For all outcomes, present, for each study: (a) summary statistics for each group (where appropriate) and (b) an effect estimate and its precision (e.g. confidence/credible interval), ideally using structured tables or plots.	Figure 2-7
Results of syntheses	20a	For each synthesis, briefly summarise the characteristics and risk of bias among contributing studies.	Supp Material 2
	20b	Present results of all statistical syntheses conducted. If meta-analysis was done, present for each the summary estimate and its precision (e.g. confidence/credible interval) and measures of statistical heterogeneity. If comparing groups, describe the direction of the effect.	Page 16-19; Table 2
	20c	Present results of all investigations of possible causes of heterogeneity among study results.	Page 16-19
	20d	Present results of all sensitivity analyses conducted to assess the robustness of the synthesized results.	Page 16-18
Reporting biases	21	Present assessments of risk of bias due to missing results (arising from reporting biases) for each synthesis assessed.	Supp Material 2
Certainty of evidence	22	Present assessments of certainty (or confidence) in the body of evidence for each outcome assessed.	Supp Material 7
**DISCUSSION**			

Section and Topic	Item #	Checklist item	Location where item is reported
Discussion	23a	Provide a general interpretation of the results in the context of other evidence.	Page 20-22
	23b	Discuss any limitations of the evidence included in the review.	Page 22-23
	23c	Discuss any limitations of the review processes used.	Page 22-23
	23d	Discuss implications of the results for practice, policy, and future research.	Page 20-22
**OTHER INFORMATION**			
Registration and protocol	24a	Provide registration information for the review, including register name and registration number, or state that the review was not registered.	Page 10
	24b	Indicate where the review protocol can be accessed, or state that a protocol was not prepared.	Page 10
	24c	Describe and explain any amendments to information provided at registration or in the protocol.	Page 10
Support	25	Describe sources of financial or non-financial support for the review, and the role of the funders or sponsors in the review.	Page 23-24
Competing interests	26	Declare any competing interests of review authors.	Page 23-24
Availability of data, code and other materials	27	Report which of the following are publicly available and where they can be found: template data collection forms; data extracted from included studies; data used for all analyses; analytic code; any other materials used in the review.	Page 10

From: Page MJ, McKenzie JE, Bossuyt PM, Boutron I, Hoffmann TC, Mulrow CD, et al. The PRISMA 2020 statement: an updated guideline for reporting systematic reviews. BMJ 2021;372:n71. doi: 10.1136/bmj.n71

For more information, visit: http://www.prisma-statement.org/

Table S4. GRADE Evidence Table

Certainty assessment							No. of patients		Effect		Certainty	Importance
No. of studies	Study design	Risk of bias	Inconsistency	Indirectness	Imprecision	Other considerations	Care at CACs	Care at non-CACs	Relative (95% CI)	Absolute (95% CI)		
Survival with good neurological outcome												Critical
5	Observational cohort studies	Not serious	Not serious	Not serious	Not serious	All plausible residual confounding reduces demonstrated effect*	1561 / 21735 (7.2%)	480 / 31237 (1.5%)	**OR 1.85** (1.52 to 2.26)	13 more per 1,000 (from 8 more to 19 more)	⊕⊕⊕◯ Moderate	
Survival with good neurological outcome, shockable rhythm subgroup												Critical
5	Observational cohort studies	Serious†	Not serious	Not serious	Not serious	Strong association‡	213 / 1376 (15.5%)	54 / 544 (9.9%)	**OR 2.47** (1.88 to 3.25)	115 more per 1,000 (from 72 more to 164 more)	⊕⊕◯◯ Low	
Survival with good neurological outcome, nonshockable rhythm subgroup												Critical
2	Observational cohort studies	Serious†	Not serious	Not serious	Not serious	-	-	-	**OR 1.43** (1.04 to 1.98)	-	⊕◯◯◯ Very low	
Survival												Critical
7	Observational cohort studies	Not serious	Not serious	Not serious	Not serious	All plausible residual confounding reduces demonstrated effect*	2536 / 13441 (18.9%)	987 / 12454 (7.9%)	**OR 1.92** (1.59 to 2.32)	63 more per 1,000 (from 41 more to 87 more)	⊕⊕⊕◯ Moderate	
Survival, prehospital ROSC subgroup												Critical
7	Observational cohort studies	Serious†	Not serious	Not serious	Not serious	-	2065 / 6200 (33.3%)	817 / 2601 (31.4%)	**OR 1.46** (1.12 to 1.90)	87 more per 1,000 (from 25 more to 151 more)	⊕◯◯◯ Very low	
Survival, no prehospital ROSC subgroup												Critical
5	Observational cohort studies	Serious†	Not serious	Not serious	Not serious	Strong association‡	412 / 3895 (10.6%)	176 / 8929 (2.0%)	**OR 2.52** (1.90 to 3.35)	29 more per 1,000 (from 17 more to 43 more)	⊕⊕◯◯ Low	

CAC: Cardiac arrest centre; OR: Odds ratio; CI: Confidence interval; ROSC: Return of spontaneous circulation

* Some studies (e.g., Mumma. et al, Harnod. et al) did not adjust for certain prehospital variables, leading to residual confounding. However, it is likely that emergency medical services transported sicker patients who had more severe prehospital characteristics to CACs for advanced care. This was expected to diminish the intervention effect.

† Inclusion of before-and-after study designs ‡ RR > 2.0 from direct evidence

Figure S1. Forest Plots for Meta-Analyses of Unadjusted Odds Ratios

1: Forest plot for meta-analysis of unadjusted analyses comparing survival with good neurological outcome between CACs and non-CACs, using a random effects model and the “strict” definition of CACs

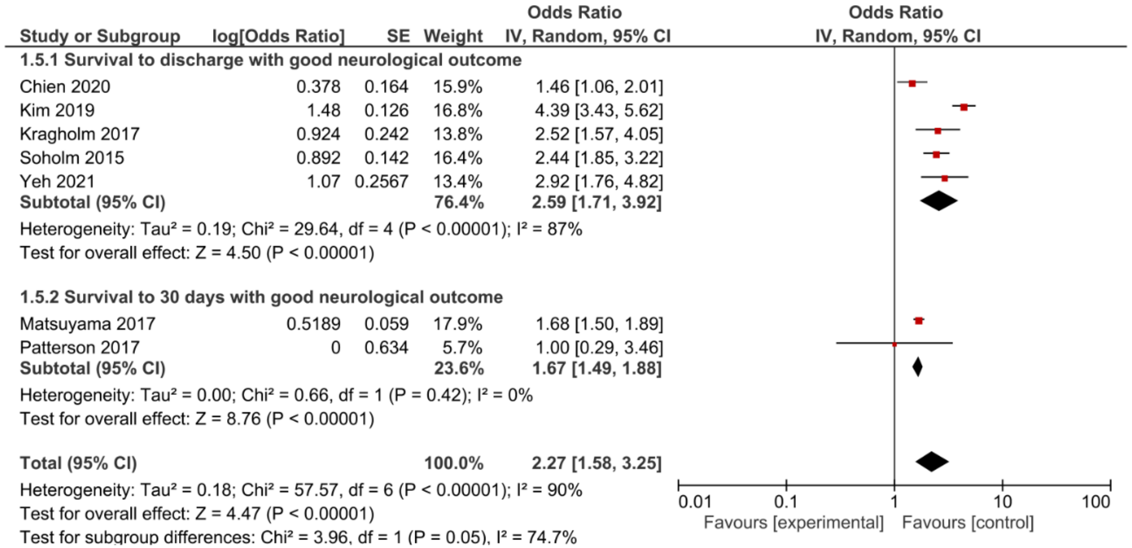

2: Forest plot for meta-analysis of unadjusted analyses comparing survival with good neurological outcome between CACs and non-CACs, using a random effects model and including high volume centres

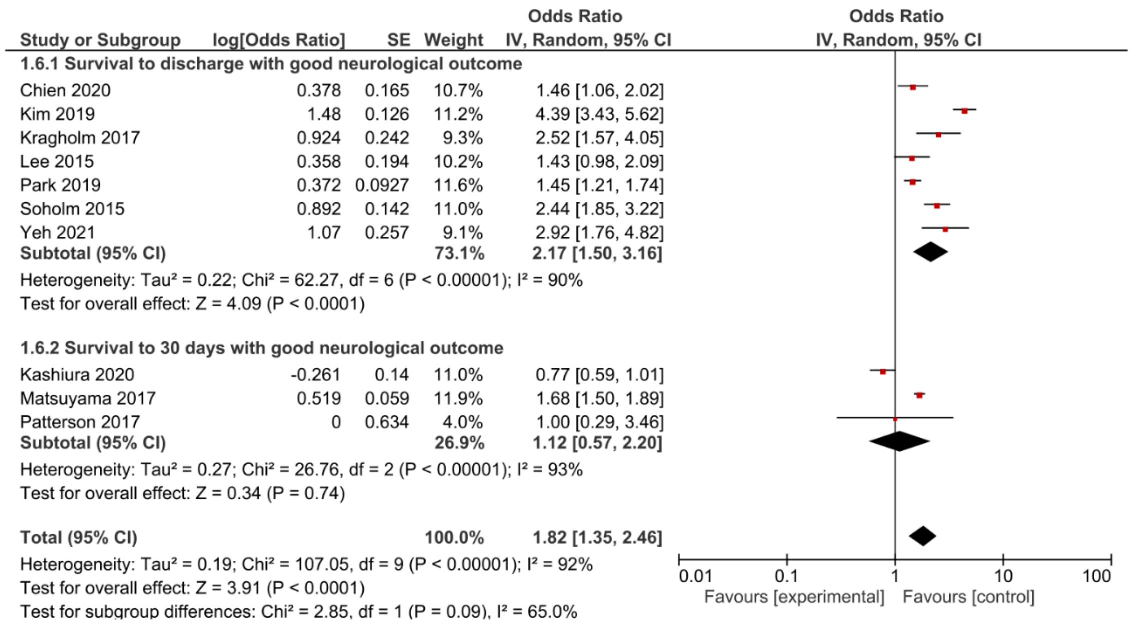

3. Forest plot for meta-analysis of unadjusted analyses comparing survival with good neurological outcome between CACs and non-CACs, using a random effects model and including improved care centres

Study or Subgroup	log[Odds Ratio]	SE	Weight	Odds Ratio IV, Random, 95% CI
**1.7.1 Survival to discharge with good neurological outcome**				
Chien 2020	0.378	0.165	9.4%	1.46 [1.06, 2.02]
Gajeski 2009	1.03	0.738	2.5%	2.80 [0.66, 11.90]
Kim 2013	0.647	0.137	9.8%	1.91 [1.46, 2.50]
Kim 2019	1.48	0.126	10.0%	4.39 [3.43, 5.62]
Kragholm 2017	0.924	0.242	8.0%	2.52 [1.57, 4.05]
Soholm 2015	0.892	0.142	9.7%	2.44 [1.85, 3.22]
Spaite 2014	0.438	0.223	8.4%	1.55 [1.00, 2.40]
Sunde 2007	1.28	0.396	5.6%	3.60 [1.66, 7.82]
Yeh 2021	1.07	0.257	7.8%	2.92 [1.76, 4.82]
Youn 2013	1.17	0.48	4.5%	3.22 [1.26, 8.25]
**Subtotal (95% CI)**			**75.6%**	**2.44 [1.84, 3.25]**
Heterogeneity: Tau² = 0.14; Chi² = 40.62, df = 9 (P < 0.00001); I² = 78%				
Test for overall effect: Z = 6.15 (P < 0.00001)				
**1.7.2 Survival to 30 days with good neurological outcome**				
Matsuyama 2017	0.519	0.059	10.7%	1.68 [1.50, 1.89]
Patterson 2017	0	0.634	3.1%	1.00 [0.29, 3.46]
Seiner 2018	-0.416	0.316	6.8%	0.66 [0.36, 1.23]
Tagami 2012	1.76	0.546	3.8%	5.81 [1.99, 16.95]
**Subtotal (95% CI)**			**24.4%**	**1.51 [0.74, 3.09]**
Heterogeneity: Tau² = 0.38; Chi² = 14.45, df = 3 (P = 0.002); I² = 79%				
Test for overall effect: Z = 1.13 (P = 0.26)				
**Total (95% CI)**			**100.0%**	**2.16 [1.67, 2.81]**
Heterogeneity: Tau² = 0.16; Chi² = 78.51, df = 13 (P < 0.00001); I² = 83%				
Test for overall effect: Z = 5.80 (P < 0.00001)				
Test for subgroup differences: Chi² = 1.49, df = 1 (P = 0.22), I² = 32.9%				

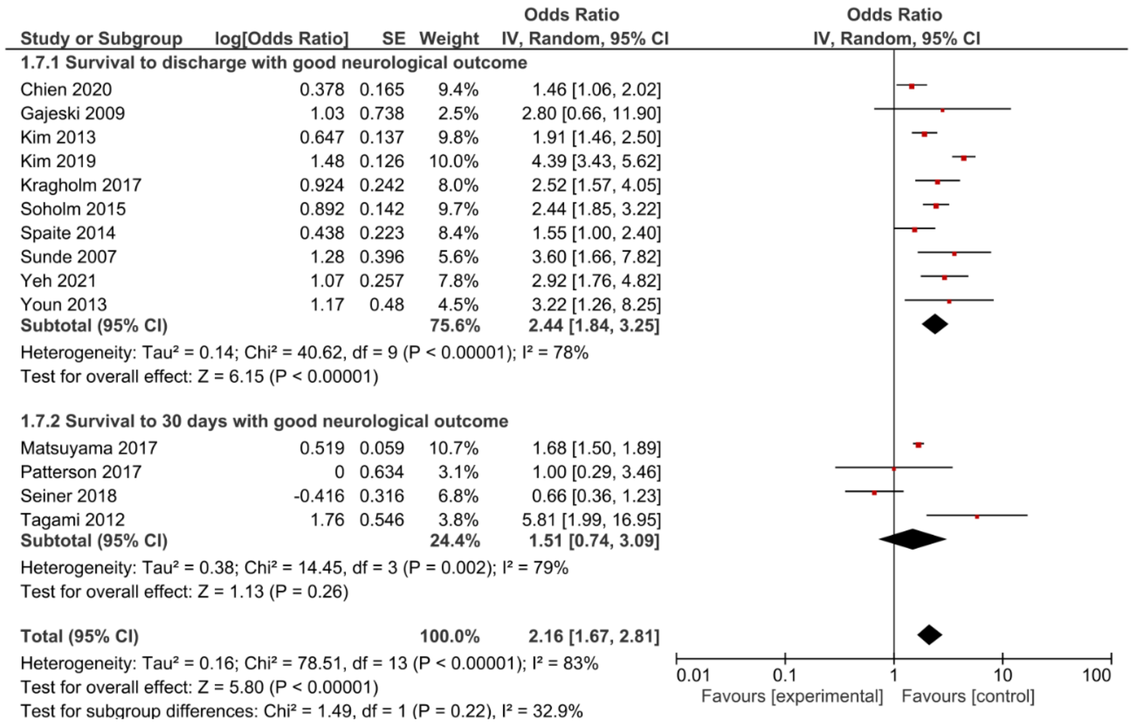

4. Forest plot for meta-analysis of unadjusted analyses comparing survival between CACs and non-CACs, using a random effects model and the "strict" definition of CACs

Study or Subgroup	log[Odds Ratio]	SE	Weight	Odds Ratio IV, Random, 95% CI
**2.5.1 Survival to discharge**				
Cournoyer 2018	0.693	0.0811	10.5%	2.00 [1.71, 2.34]
Kim 2019	1.3	0.088	10.3%	3.67 [3.09, 4.36]
Kragholm 2017	1.08	0.242	6.8%	2.94 [1.83, 4.73]
Lai 2018	0.798	0.0698	10.6%	2.22 [1.94, 2.55]
Soholm 2015	0.978	0.135	9.3%	2.66 [2.04, 3.46]
Stub 2011	0.344	0.0916	10.3%	1.41 [1.18, 1.69]
Vopelius-Feldt 2021	0.392	0.121	9.7%	1.48 [1.17, 1.88]
Yeh 2021	0.761	0.184	8.2%	2.14 [1.49, 3.07]
**Subtotal (95% CI)**			**75.7%**	**2.19 [1.72, 2.78]**
Heterogeneity: Tau² = 0.10; Chi² = 72.73, df = 7 (P < 0.00001); I² = 90%				
Test for overall effect: Z = 6.40 (P < 0.00001)				
**2.5.2 Survival to 30 days**				
Kajino 2010	0.837	0.0782	10.5%	2.31 [1.98, 2.69]
Patterson 2017	-0.511	0.384	4.3%	0.60 [0.28, 1.27]
Soholm 2013	1.08	0.127	9.5%	2.94 [2.30, 3.78]
**Subtotal (95% CI)**			**24.3%**	**1.92 [1.19, 3.09]**
Heterogeneity: Tau² = 0.14; Chi² = 15.83, df = 2 (P = 0.0004); I² = 87%				
Test for overall effect: Z = 2.68 (P = 0.007)				
**Total (95% CI)**			**100.0%**	**2.14 [1.75, 2.61]**
Heterogeneity: Tau² = 0.09; Chi² = 90.00, df = 10 (P < 0.00001); I² = 89%				
Test for overall effect: Z = 7.51 (P < 0.00001)				
Test for subgroup differences: Chi² = 0.23, df = 1 (P = 0.63), I² = 0%				

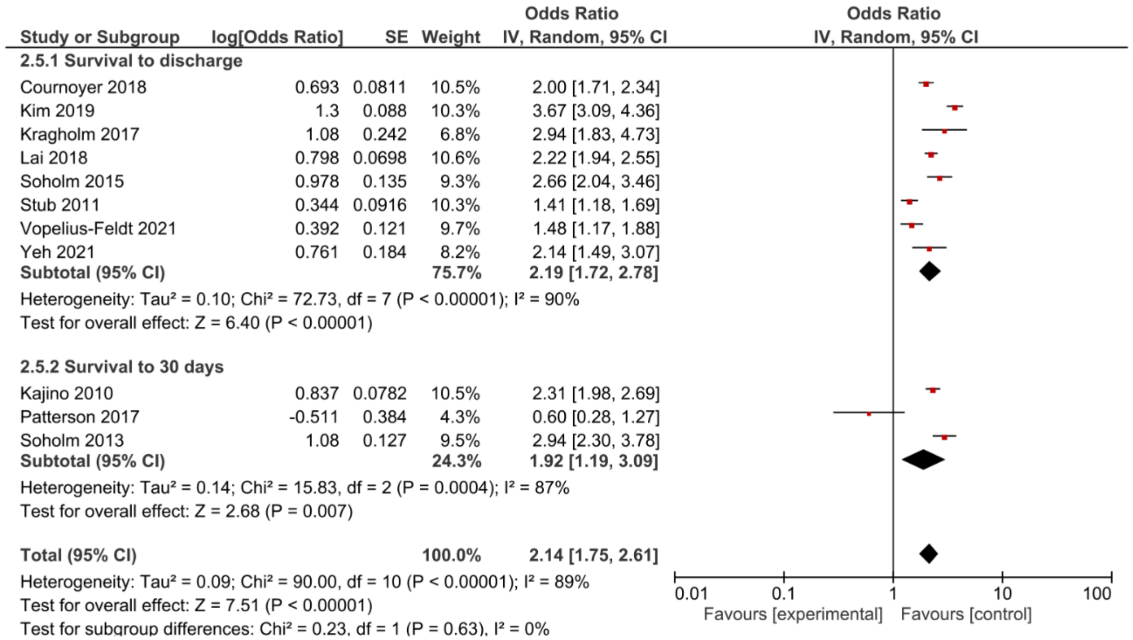

5. Forest plot for meta-analysis of unadjusted analyses comparing survival between CACs and non-CACs, using a random effects model and including high volume centres

Study or Subgroup	log[Odds Ratio]	SE	Weight	Odds Ratio IV, Random, 95% CI
**2.6.1 Survival to discharge**				
Cha 2012	1.24	0.08	6.1%	3.46 [2.95, 4.04]
Chocron 2017	-0.0619	0.202	5.0%	0.94 [0.63, 1.40]
Cournoyer 2018	0.693	0.0811	6.0%	2.00 [1.71, 2.34]
Kim 2019	1.3	0.0882	6.0%	3.67 [3.09, 4.36]
Kragholm 2017	1.08	0.242	4.6%	2.94 [1.83, 4.73]
Lai 2018	0.798	0.0698	6.1%	2.22 [1.94, 2.55]
Lee 2015	0.174	0.169	5.3%	1.19 [0.85, 1.66]
Park 2019	0.191	0.0777	6.1%	1.21 [1.04, 1.41]
Shin 2011	0.536	0.116	5.8%	1.71 [1.36, 2.15]
Soholm 2015	0.978	0.135	5.6%	2.66 [2.04, 3.46]
Stub 2011	0.344	0.0916	6.0%	1.41 [1.18, 1.69]
Vopelius-Feldt 2021	0.392	0.121	5.8%	1.48 [1.17, 1.88]
Yeh 2021	0.761	0.184	5.2%	2.14 [1.49, 3.07]
**Subtotal (95% CI)**			**73.6%**	**1.92 [1.52, 2.42]**
Heterogeneity: Tau² = 0.16; Chi² = 191.00, df = 12 (P < 0.00001); I² = 94%				
Test for overall effect: Z = 5.53 (P < 0.00001)				
**2.6.2 Survival to 30 days**				
Kajino 2010	0.837	0.0782	6.1%	2.31 [1.98, 2.69]
Kashiura 2020	1.3	0.0882	6.0%	3.67 [3.09, 4.36]
Patterson 2017	-0.511	0.384	3.2%	0.60 [0.28, 1.27]
Schober 2016	0.519	0.163	5.4%	1.68 [1.22, 2.31]
Soholm 2013	1.08	0.127	5.7%	2.94 [2.30, 3.78]
**Subtotal (95% CI)**			**26.4%**	**2.18 [1.53, 3.09]**
Heterogeneity: Tau² = 0.13; Chi² = 40.17, df = 4 (P < 0.00001); I² = 90%				
Test for overall effect: Z = 4.35 (P < 0.0001)				
**Total (95% CI)**			**100.0%**	**1.98 [1.63, 2.40]**
Heterogeneity: Tau² = 0.16; Chi² = 252.39, df = 17 (P < 0.00001); I² = 93%				
Test for overall effect: Z = 6.85 (P < 0.00001)				
Test for subgroup differences: Chi² = 0.34, df = 1 (P = 0.56), I² = 0%				

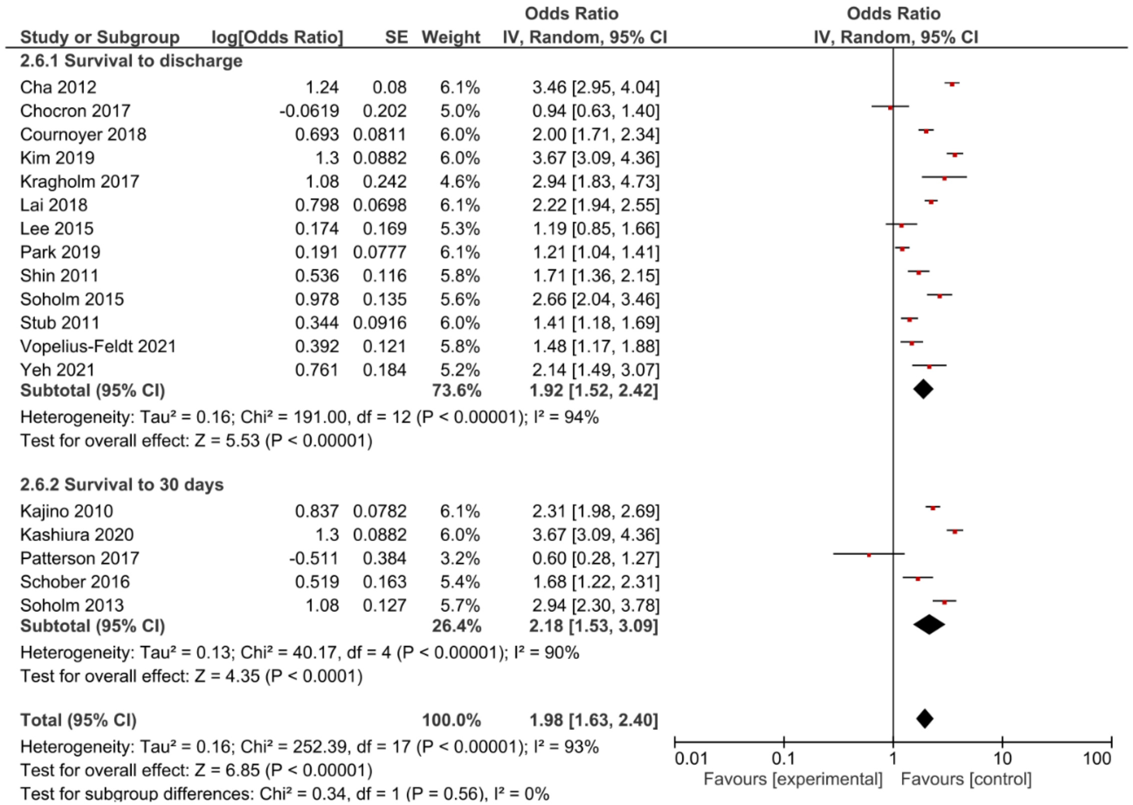

6. Forest plot for meta-analysis of unadjusted analyses comparing survival between CACs and non-CACs, using a random effects model and including improved care centres

Study or Subgroup	log[Odds Ratio]	SE	Weight	Odds Ratio IV, Random, 95% CI
**2.7.1 Survival to discharge**				
Cournoyer 2018	0.693	0.0811	7.5%	2.00 [1.71, 2.34]
Gajeski 2009	1.25	0.736	1.2%	3.49 [0.82, 14.77]
Kim 2019	1.3	0.0882	7.4%	3.67 [3.09, 4.36]
Kragholm 2017	1.08	0.242	5.0%	2.94 [1.83, 4.73]
Lai 2018	0.798	0.0698	7.6%	2.22 [1.94, 2.55]
Lick 2011	0.956	0.424	2.8%	2.60 [1.13, 5.97]
Soholm 2015	0.978	0.135	6.8%	2.66 [2.04, 3.46]
Spaite 2014	0.525	0.184	6.0%	1.69 [1.18, 2.42]
Stub 2011	0.344	0.0916	7.4%	1.41 [1.18, 1.69]
Sunde 2007	1.03	0.383	3.2%	2.80 [1.32, 5.93]
Vopelius-Feldt 2021	0.392	0.121	7.0%	1.48 [1.17, 1.88]
Walters 2011	0.604	0.567	1.9%	1.83 [0.60, 5.56]
Yeh 2021	0.761	0.184	6.0%	2.14 [1.49, 3.07]
Youn 2013	0.886	0.315	4.0%	2.43 [1.31, 4.50]
**Subtotal (95% CI)**			**73.7%**	**2.20 [1.81, 2.67]**
Heterogeneity: Tau² = 0.09; Chi² = 75.65, df = 13 (P < 0.00001); I² = 83%				
Test for overall effect: Z = 7.91 (P < 0.00001)				
**2.7.2 Survival to 30 days**				
Kajino 2010	0.837	0.0782	7.5%	2.31 [1.98, 2.69]
Patterson 2017	-0.511	0.383	3.2%	0.60 [0.28, 1.27]
Seiner 2018	-0.58	0.306	4.1%	0.56 [0.31, 1.02]
Soholm 2013	1.08	0.127	6.9%	2.94 [2.30, 3.78]
Tagami 2012	0.779	0.274	4.5%	2.18 [1.27, 3.73]
**Subtotal (95% CI)**			**26.3%**	**1.52 [0.94, 2.46]**
Heterogeneity: Tau² = 0.24; Chi² = 37.14, df = 4 (P < 0.00001); I² = 89%				
Test for overall effect: Z = 1.70 (P = 0.09)				
**Total (95% CI)**			**100.0%**	**2.04 [1.72, 2.43]**
Heterogeneity: Tau² = 0.10; Chi² = 112.96, df = 18 (P < 0.00001); I² = 84%				
Test for overall effect: Z = 8.03 (P < 0.00001)				
Test for subgroup differences: Chi² = 1.92, df = 1 (P = 0.17), I² = 47.8%				

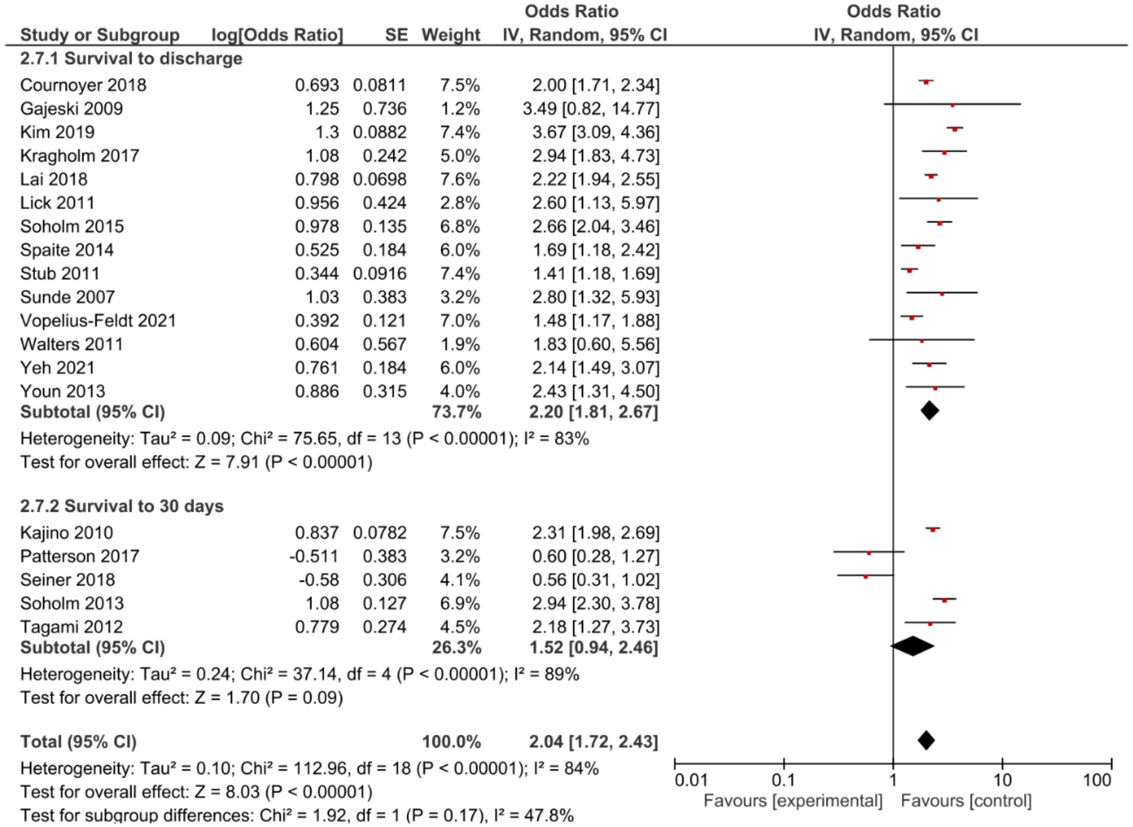

Figure S2. Funnel Plots

1: Survival to discharge or 30 days with good neurological outcome (CACs and improved care centers)

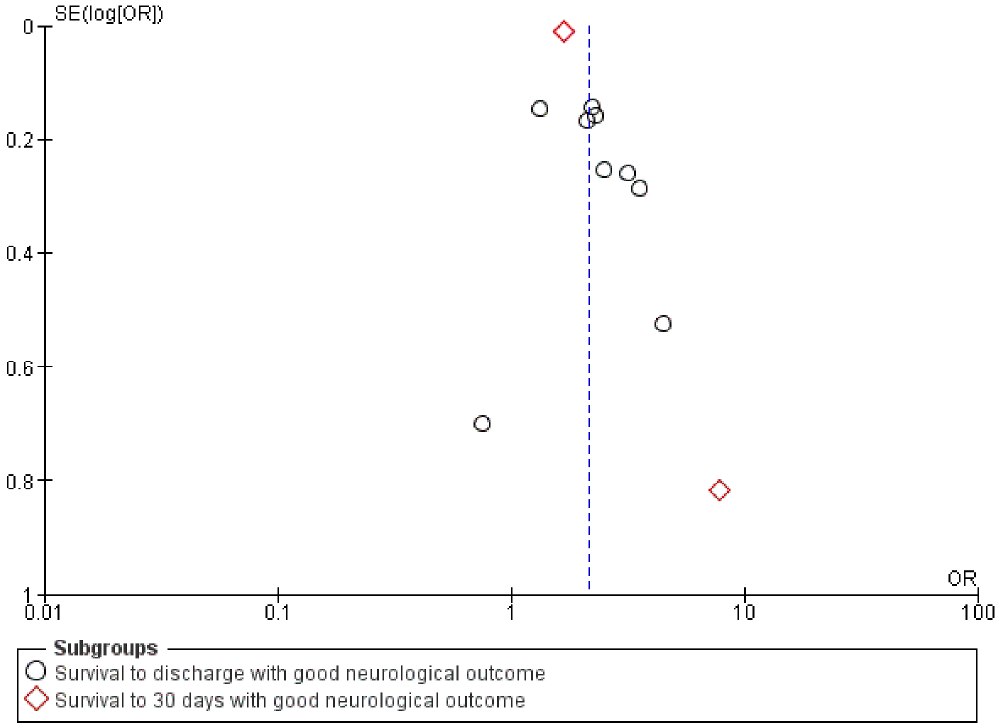

2: Survival to discharge or 30 days (CACs and improved care centers)

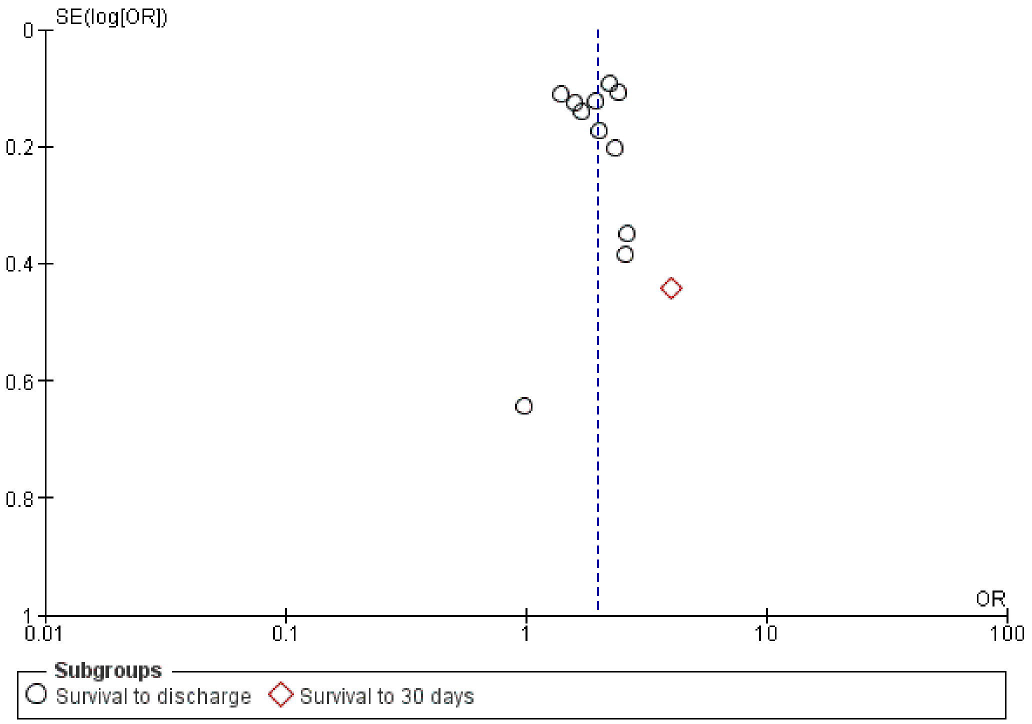

SE(log[OR])
0
0.2
0.4
0.6
0.8
1
OR
0.01
0.1
1
10
100
Subgroups
Survival to discharge
Survival to 30 days
